# Harnessing Atomically Dispersed Cobalt for the Reductive Catalytic Fractionation of Lignocellulose

**DOI:** 10.1002/advs.202310202

**Published:** 2024-03-17

**Authors:** Xiancheng Li, Rumin Ma, Xueying Gao, Helong Li, Shuizhong Wang, Guoyong Song

**Affiliations:** ^1^ State Key Laboratory of Efficient Production of Forest Resources Beijing Key Laboratory of Lignocellulosic Chemistry Beijing Forestry University Beijing 100083 China; ^2^ Institute of Nuclear and New Energy Technology Tsinghua University Beijing 100084 China

**Keywords:** atomically dispersed cobalt, hydrogenolysis, lignin, lignocellulose, reductive catalytic fractionation

## Abstract

The reductive catalytic fractionation (RCF) of lignocellulose, considering lignin valorization at design time, has demonstrated the entire utilization of all lignocellulose components; however, such processes always require catalysts based on precious metals or high‐loaded nonprecious metals. Herein, the study develops an ultra‐low loaded, atomically dispersed cobalt catalyst, which displays an exceptional performance in the RCF of lignocellulose. An approximately theoretical maximum yield of phenolic monomers (48.3 wt.%) from lignin is realized, rivaling precious metal catalysts. High selectivity toward 4‐propyl‐substituted guaiacol/syringol facilitates their purification and follows syntheses of highly adhesive polyesters. Lignin nanoparticles (LNPs) are generated by simple treatment of the obtained phenolic dimers and oligomers. RCF‐resulted carbohydrate pulp are more obedient to enzymatic hydrolysis. Experimental studies on lignin model compounds reveal the concerted cleavage of C_α_–O and C_β_–O pathway for the rupture of β‐O‐4 structure. Overall, the approach involves valorizing products derived from lignin biopolymer, providing the opportunity for the comprehensive utilization of all components within lignocellulose.

## Graphical abstract

An ultra‐low loaded, atomically dispersed cobalt demonstrates exceptional catalytic performance in the reductive catalytic fractionation of lignocellulose. Such a process 1) maximizes the theoretical yield of phenolic monomers suitable for synthesizing highly adhesive polyesters, 2) produces phenolic dimers and oligomers feedstocks for preparing lignin nanoparticles, and 3) preserves cellulose and hemicellulose amenable to enzymatic hydrolysis.

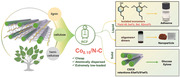

## 1 Introduction

**Figure 1:**
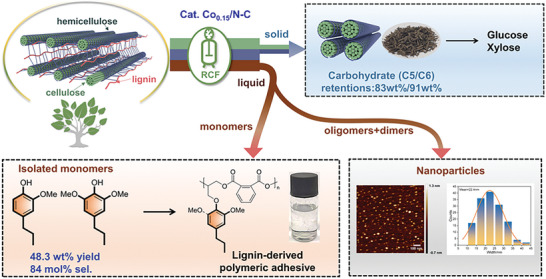
Schematic flow diagram of reductive catalytic fractionation of lignocellulose.

The concerns on the unrestricted depletion of fossil fuels that has triggered excessive CO_2_ emissions drive the development of alternative and sustainable feedstocks for producing energy, chemicals, and materials.^[^ ^1^, ^2^, ^3^ ^]^ Lignocellulosic biomass is a bountiful nonedible, plant‐based green carbon resource, which is realized as a promising alternative/complement to fossil resources.^[^ ^4^, ^5^, ^6^ ^]^ Such a renewable feedstock is composed of three interlinked biopolymers, i.e., cellulose (40‐60%), hemicellulose (10‐30%), and lignin (15–30%) (**Figure** **1**).^[^ ^7^ ^]^ Most of the lignocellulosic biorefinery paradigms that have been employed in the past and at present focused on the conversion of cellulose and hemicellulose to biofuels and biomaterials,^[^ ^4^, ^7^, ^8^ ^]^ and instated lignin, the high energy density aromatic biopolymer, for heat and power production.^[^ ^6^, ^9^ ^]^ Therefore, the key to advancing biorefinery economics and enabling the fullest utilization of lignocellulose is the valorization of lignin.^[^ ^10^ ^]^ Lignin biopolymers are primarily composed of three phenylpropanoid groups, that is syringyl (S), guaiacyl (G), and hydroxyphenyl (H) units, being connected by C–O (such as β‐O‐4) and C–C (such as β−5 and β‐β) type linkages.^[^ ^1^, ^11^, ^12^ ^]^ As lignin represents the largest renewable aromatic resource in nature, it has significant potential to serve as a feedstock to produce bulk or functionalized aromatic compounds;^[^ ^13^ ^]^ however, the valorization of lignin has remained a scientifically intriguing research problem spanning the last century, due to the challenges associated with isolating lignin from biomass matrices without recondensation and the difficulty in overcoming the inherent heterogeneity of lignin.

The “lignin‐first” biorefining approach, where lignin in biomass matrix is preferentially extracted and depolymerized, together with the preservation of (hemi)cellulose biopolymers, has demonstrated the fullest utilization of lignocellulose.^[^ ^14^ ^]^ This approach is generally carried out in the presence of a transition metal catalyst under a reduced atmosphere, being termed as reductive catalytic fractionation (RCF).^[^ ^15^, ^16^, ^17^ ^]^ The transition metal catalysts, which dominate the cleavage of lignin linkages and subsequent stabilization steps, have been evolved rapidly in the last decade. In early period, commercial catalysts with high‐loaded (*ca*. 5 wt.% loading) and nanostructured precious metal centers, such as Ru/C,^[^ ^18^, ^19^, ^20^ ^]^ Pd/C,^[^ ^21^, ^22^ ^]^ Pt/C,^[^ ^23^ ^]^ and Rh/C,^[^ ^24^ ^]^ were used in the RCF of lignocelluloses. To avoid the use of precious metals, some catalysts having high‐loaded nonprecious metals (5‐33 wt.% loading), including Ni,^[^ ^25^, ^26^ ^]^ Mo,^[^ ^27^ ^]^ Cu,^[^ ^28^, ^29^ ^]^ and W,^[^ ^30^ ^]^ have been proposed for the RCF of woods and grasses. Inspired by the merits of single‐atom catalysts (SACs) of ≈100% metal atomic utilization and unique electronic structures,^[^ ^31^, ^32^ ^]^ several low‐loaded, atomically dispersed metal catalysts were recently developed, which have been proven to be effective in the RCF of lignocelluloses. For example, SACs based on Pd,^[^ ^33^, ^34^ ^]^ Pt^[^ ^23^ ^]^ and Ru^[^ ^35^, ^36^ ^]^ catalysts (0.18–0.3 wt.% metal loading) enabled the formation of phenolic monomers in approximately theoretical maximum yields, with the improvement of the turnover numbers (TONs = 166–431 mol_phenols_ mol_active metal_^−1^) by one order of magnitude as compared with commercial Pd/C, Pt/C, and Ru/C catalysts (TONs = 10–30 mol_phenols_ mol_active metal_^−1^).^[^ ^35^ ^]^ From a practical aspect, engineering a SACs based on nonprecious metal centers for lignocellulose RCF, which feature catalytic performance comparable to those precious metal catalysts, regarding the yield and selectivity of monophenols, the retentions of carbohydrate, and the utilization efficiency of active metal centers, would be highly desired but challenging.

Herein, we demonstrated an ultra‐low loaded, atomically dispersed, and *N*‐coordinated Co catalyst Co_0.15_/N‐C (0.15 wt.% Co loading) synthesized via a cascade anchoring strategy and followed thermal treatment.^[^ ^37^ ^]^ This catalyst was very effective for the RCF of lignocelluloses, which delivered phenolic monomers in theoretical maximum yield (48 wt.%) with a high selectivity (84%) to propyl‐substituted syringol (**S1**) and guaiacol (**G1**), and left solid cellulose and hemicellulose with 91 and 83 wt.% retentions. The Co_0.15_/N‐C catalyst displayed good stability in recycling experiments as well as after a hydrothermal treatment. Systematical experiments by scanning the reactivity of various β‐O‐4 models provided inspiring insights into the mechanism of lignin hydrogenolysis over Co_0.15_/N‐C catalyst. Almost all components in lignocellulose can be valorized into chemicals and materials after current RCF treatment (Figure 1): i) the high selectivity of **S1/G1** allowed them to be purified from reaction mixture readily, which were subsequently functionalized and copolymerized into a new family of polyesters intended as strong adhesives; ii) the lignin‐derived dimers and oligomers steam were precipitated in the form of uniformed nanoparticles (NPs); iii) the carbohydrate‐rich solid fraction can be efficiently transformed into sugars through enzymatic hydrolysis owing to the successful breaking biomass recalcitrance.

## 2 Results

### 2.1 Preparation and Characterization of Co_0.15_/N‐C Catalyst

**Figure 2:**
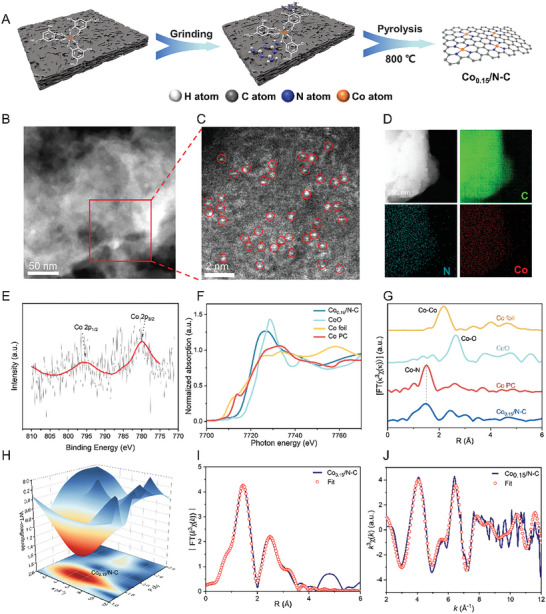
Synthesis and structure characterization of Co_0.15_/N‐C. A) Schematic flow illustration of the fabrication of Co_0.15_/N‐C. B,C) HAADF‐STEM images of Co_0.15_/N‐C. D) EDX element mapping image of Co_0.15_/N‐C. E) Co 2p XPS spectra of Co_0.15_/N‐C, F) Co K‐edge XANES of Co_0.15_/N‐C, and reference samples, G) FT‐EXAFS, and H) WT‐EXAFS of Co_0.15_/N‐C. EXAFS fitting curves of Co_0.15_/N‐C in I) *R* space and J) *k*‐space.

The Co_0.15_/N‐C catalyst was prepared via a cascade anchoring strategy,^[^ ^37^ ^]^ which included: 1) loading the complexion of Co(NO_3_)_2_ and gallic acid (*O*‐bonded Co species) on activated carbon; 2) mixing the complex‐loaded carbon with melamine (a nitrogen source) by grinding; 3) forming *N*‐bonded Co species via pyrolysis under nitrogen at 800 °C. The use of gallic acid as a chelate ligand can avoid the aggregation of Co ions during loading process, and the introduction of nitrogen species could stabilize and increase the electron density of the Co centers through *N*‐coordination (**Figure** **2**A).

In the resultant Co_0.15_/N‐C catalyst, the Co content was determined to be 0.15 wt.% by inductively coupled plasma optical emission spectroscopy (ICP‐OES) (Table S1, Supporting Information). Brunauer–Emmett–Teller (BET) adsorption‐desorption isotherms implied that this catalyst featured microporous structures, with a specific surface area of 793 m^2^ g^−1^ and a pore volume of 0.63 cm^3^ g^−1^ (Figure S1 and Table S1, Supporting Information). The powder X‐ray diffraction (XRD) patterns revealed two broad peaks ascribed to the activated carbon; there were no detectable Co‐related peaks, probably because of the ultra‐low content and/or high dispersion of Co species (Figure S2, Supporting Information).^[^ ^38^ ^]^ In aberration‐corrected high‐angle annular dark‐field scanning transmission electron microscopy (HADDF‐STEM) images, a large number of atom‐sized bright spots were assigned to single Co atoms, with no Co nanoparticles or small clusters observed (Figure 2B,C). Energy‐dispersive X‐ray spectroscopy (EDX) mapping implied that the elements of Co, C, and N were uniformly distributed on the catalyst surface (Figure 2D). These results indicated the isolated dispersion of Co atoms on the carbon support. X‐ray photoelectron spectroscopy (XPS) analysis indicated the presence of Co, C, and N species in the Co_0.15_/N‐C catalyst, with characteristic peaks for C 1s at 284.7 eV (C═C), 285.7 eV (C═N), 286.6 eV (C–N), and 289.8 eV (C═O),^[^ ^33^ ^]^ as well as N 1s peaks at 398.2 eV (pyridinic N), 399.9 eV (pyrrolic N), 401.0 eV (graphitic N), and 403.4 eV (oxidized N) (Figure S3, Supporting Information). In XPS results, the binding energy of the Co 2p peaks resonated at 796.2 and 780.2 eV, implying the positive valence feature of Co single atoms (Figure 2E).^[^ ^39^ ^]^

The electronic structure of Co species was further measured by the X‐ray absorption near‐edge structure (XANES) and Fourier transform extended X‐ray absorption fine structure (FT‐EXAFS). In the XANES spectra, the curve of Co in Co_0.15_/N‐C situated between those of CoO and Co foil, implying a positive valence state (Figure 2F), being well consistent with the XPS results. The FT‐EXAFS spectrum showed that Co_0.15_/N‐C exhibited a strong peak at 1.47 Å, corresponding to Co–N scattering. No Co–Co or Co–O coordination peaks were observed compared with Co foils and CoO, demonstrating that Co species were dispersed in an atomic version (Figure 2G). The wavelet transform (WT) contour plots of Co_0.15_/N‐C exhibited the only one Co–N interaction intensity maximum at 4.71Å^−1^, without the scattering paths of Co‐Co or Co–O (Figure 2H; Figure S4, Supporting Information). These XAFS results confirmed the single‐atomic dispersion of Co sites, which coincided with the observation in HADDF‐STEM images. The Co coordination number was calculated as *ca*. 4, and the average Co–N/C bond length was 1.96 Å, indicating that each Co atom is anchored by 4 N/C coordination within the *N*‐doped carbon matrix (Table S2, Supporting Information). The EXAFS fitting curves in *R*‐space (Figure 2I) and *k*‐space (Figure 2J) fitted well with the experimental curves, indicating the successful establishment of the Co–N/C_4_ configuration in Co_0.15_/N‐C.

### 2.2 Lignin‐Derived Products from RCF of Silver Birch

Hardwood (such as birch, eucalyptus, and poplar) lignin are composed of syringyl (S) and guaiacyl (G) units, being connected by abundant β‐O‐4 linkages. A silver birch (*Betula pendula* Roth) was selected for RCF experiment over Co_0.15_/N‐C.^[^ ^40^ ^]^ The contents of cellulose, hemicellulose, and lignin were determined as 42.3%, 18.1%, and 25.3% by weight based on the biomass composition analysis (Table S3, Supporting Information). To decode the chemical structure of lignin in this silver birch, a lignin sample was isolated through the combination treatment of enzymatic hydrolysis and mild acidolysis extraction (EMAL). GPC data gave an 8400 g mol^−1^ average molecular weight of this lignin. Based on semi‐quantitative analysis of the HSQC cross‐peak intensities, the S and G subunits was established as 3.7:1. The abundance of β‐O‐4 linkages was measured as *ca*. 70%, which demonstrated a *ca*. 49% of the theoretical maximum yield of phenolic monomers during lignin depolymerization (Figure S6, Supporting Information).^[^ ^35^ ^]^

**Figure 3:**
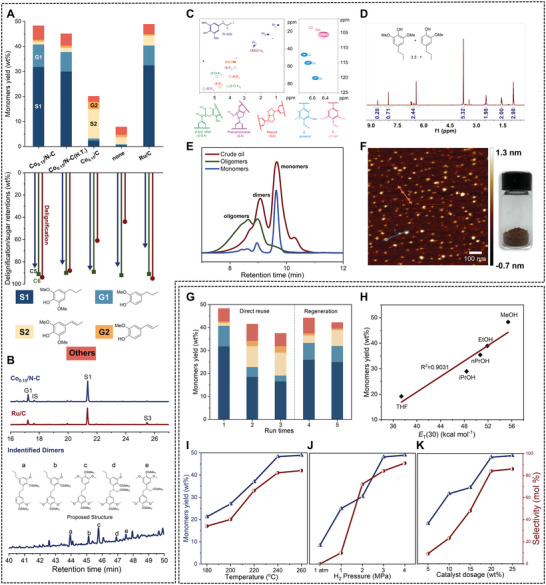
Lignin‐derived products from RCF of silver birch. A) Comparisons of phenolic monomer yields, delignification degrees and C5/C6 retentions over different catalysts. B) GC spectra of the lignin‐derived monomers and phenolic dimers. C) 2D HSQC NMR spectra of lignin‐derived products from RCF of birch over Co_0.15_/N‐C. D) ^1^H NMR spectrum of mixture of G1 and S1 after short column chromatography. E) GPC spectra of raw biomass depolymerization products after extraction with hexane. F) AFM images of LNPs. G) Stability and reusability of Co_0.15_/N‐C catalysts. Influences of H) solvents, I) temperature, J) hydrogen pressure, K) catalyst dosage for the catalytic hydrogenolysis of birch sawdust with Co_0.15_/N‐C. Reaction conditions: birch wood (250 mg), catalyst (50 mg, 20 wt.%), MeOH (10 mL), 240 °C, H_2_ (3 MPa at 25 °C, 12 MPa at 240 °C), and 4 h.

The RCF of silver birch (sawdust) was initially performed at 240 °C and 3 MPa H_2_ in MeOH, by using 20 wt.% of Co_0.15_/N‐C catalyst (where Co dose is 0.03 wt.% to birch). The 4 h reaction resulted in a soluble fraction mainly containing lignin‐derived products, and a solid fraction having cellulose (C6 sugar), hemicellulose (C5 sugar), and the catalyst. The average molecular weight (*M*_w_) of lignin‐derived products was measured as 323 g mol^−1^, being far lower than that of the isolated lignin sample, which suggested an efficient depolymerization of lignin biopolymer. The identification and quantification of monomeric phenols were carried out on gas chromatography (GC) by comparison with corresponding authentic samples. A 48.3 wt.% combined yield of phenolic monomer was normalized based on the Klason lignin, which closely approached the maximum theoretical monomer yield (Table S4, Supporting Information). The as‐calculated TON value, 100 mol_phenols_ mol_Co_^−1^, was significantly larger than most precious and nonprecious metal catalysts.^[^ ^19^, ^27^, ^28^, ^29^ ^]^ Among the monomers, propyl‐substituted syringol (**S1**, 31.8 wt.%) and guaiacol (**G1**, 9.0 wt.%) were identified as two major products, corresponding to 84 mol% selectivity of total monomers (**Figure** **3**A). The ratio of all detectable syringol‐ and guaiacyl‐derived monomers (S/G) was determined to be 3.4 (mol/mol), being similar to the S/G monomer composition in native lignin (3.7). Several phenolic dimers were also detected in the GC‐MS profile after trimethylsilylation of the lignin‐depolymerized products, all of which contained C−C bonds (Figure 3B; Figure S7, Supporting Information).^[^ ^19^ ^]^ The 2D‐HSQC NMR spectra of resultant lignin derivatives demonstrated the absence of the β‐O‐4 units, confirming a complete depolymerization of lignin over Co_0.15_/N‐C catalyst (Figure 3C). The emerged cross peaks resonated at *δ*_C_/*δ*_H_ = 37.0/2.43, 24.0/1.54, and 13.4/0.87 ppm were ascribed to the propyl end‐chain in **S1** and **G1**, and this scenario was in good agreement with the observation in GC profiles (Figure 3B).^[^ ^35^ ^]^

The separation and purification of monomeric phenols from complex mixtures is the crux and challenge for transforming lignin into valuable products.^[^ ^41^ ^]^ Owing to the high selectivity in current lignin depolymerization, a simple extraction with hexane enabled the isolation of monophenols, **S1** and **G1** in 3.3:1 mole ratio (Figure 3D), together leaving a solid containing lignin‐derived dimers and oligomers. This separation procedure was testified by GPC profiles (Figure 3E). Further silica gel column chromatography method allowed the complete isolation of **S1** and **G1** in a pure fashion, thus paving the way for preparing chemicals and materials from lignin monomers.

RCF‐generated phenolic dimers and oligomers account for almost half of lignin biopolymer; however, their application was rarely discussed in previous reports.^[^ ^6^, ^42^ ^]^ In this work, the as‐obtained dimeric and oligomeric faction shew up in dark red, which upon an antisolvent crystallization method, gave lignin nanoparticles (LNPs). Morphology characterizations by transmission electron microscopy (TEM) and atomic force microscopy (AFM) (Figure 3F; Figure S8, Supporting Information) confirmed that these LNPs were uniformly dispersed with an average width of 22.4 nm (Figure S9, Supporting Information). As LNPs have potential applications in drug carriers,^[^ ^43^ ^]^ antimicrobials,^[^ ^44^ ^]^ and hydrogels,^[^ ^45^ ^]^ this finding opens up a new avenue for the entire utilization of all lignin components.

### 2.3 Carbohydrate Pulp

In addition to lignin‐derived products, the RCF of silver birch synchronously resulted in solid leftovers containing carbohydrate and catalyst. A simple sieving enabled the separation of the Co_0.15_/N‐C catalyst (92 wt.% recovery) and carbohydrate, owing to the well‐remained original framework of biomass (2‐5 mm). Based on biomass compositional analyses, it was found that 94 wt.% of lignin had been removed from the biomass matrix, and 91 wt.% of cellulose (C6) and 83 wt.% of hemicellulose (C5) were reserved in the solid fraction (Table S4, Supporting Information). These results demonstrated an efficient fractionation of lignocellulose components and breaking biomass recalcitrance. Thereby, upon the treatment of carbohydrate pulp with Cellic@CTec3 enzyme, impressive yields of glucose (92%) and xylose (79%) from the hydrolysis of cellulose and hemicellulose were achieved in 72 h, respectively. In comparison, untreated ball‐milled birch sawdust gave much lower yields of glucose (41%) and xylose (19%) (Figure S10, Supporting Information). The substantial difference in sugar yields between the catalyst‐treated and untreated birch biomass highlights the effectiveness of the Co_0.15_/N‐C catalyst in promoting the valorization of carbohydrates.

### 2.4 Stability and Reusability of Co_0.15_/N‐C

The stability and reusability of a heterogeneous catalyst should be guaranteed before considering it for commercial applications. To evaluate the stability of Co_0.15_/N‐C under RCF conditions, it was treated in water at 200 °C for 72 h (Table S4, Supporting Information), thus giving a hydrothermal‐treated catalyst, Co_0.15_/N‐C (H.T.). In RCF of birch, this catalyst afforded 45.1 wt.% yield of monomeric phenols, 84% selectivity to **S1** and **G1**, and 88 wt.% of delignification degree, as well as 90 wt.% and 80 wt.% retentions of cellulose and hemicellulose, showing a similar catalytic performance to fresh catalyst (Figure 3A). These results indicated that Co_0.15_/N‐C features high tolerance against deactivation under harsh hydrothermal conditions.

The reusability of Co_0.15_/N‐C was also tested after it was isolated from carbohydrate pulp. In the case of direct using the spent Co_0.15_/N‐C (2nd and 3rd runs), declines in the monomers yields and selectivity to **S1** and **G1** were observed (Figure 3G). The ICP‐OES of analysis gave a 0.14 wt.% Co content in the spent catalyst, which was very close to the fresh catalyst (Table S1, Supporting Information). In addition, the spent Co_0.15_/N‐C presented a much lower BET surface area (198 m^2^ g^−1^) than the fresh one (793 m^2^ g^−1^). These results suggested that the reduction of catalytic performance was probably caused by the adsorption of organic species during RCF. In this context, the spent catalyst was regenerated under 600 °C for 2 h, by which the porous structures were partially resumed. In the 4th and 5th RCF runs, the regenerated Co_0.15_/N‐C resulted in 44.2 wt.% and 42.2 wt.% yields of monophenols (Table S5, Supporting Information), implying almost complete recovery of catalytic performance (Figure 3G).

### 2.5 Catalysts and Parameters Screening

For comparison, several catalysts were employed to screen the RCF of silver birch (Figure 3A). In the presence of 10 wt.% of commercial Ru/C catalyst under similar conditions, a 48.9 wt.% of aromatic monomers yield, together with 83% selectivity to **S1** and **G1**, were obtained. Due to the high loading of precious Ru (5 wt.%), the TON value was calculated as only 23, lower than that from the current nonprecious Co_0.15_/C catalyst. We also prepared an extremely low‐loaded Co catalyst Co_0.15_/C lacking *N* doping, by which significant drops in lignin monomers yield (19.9 wt.%), the degree of delignification (61 wt.%), and the selectivity to **S1** and **G1** (16%). The electron‐donated *N* species that bonded the Co atoms can enhance the activity toward the cleavage of the C−O bonds and hydrogenation of C═C bonds. The use of *N*‐doped carbon gave a poor yield of phenolic monomers (8.1 wt.%), similar to that from the catalyst‐free experiment (7.8 wt.%), demonstrating the curial role of Co species, albeit with extremely low loading (Table S4, Supporting Information).

The screening of several solvents in the presence of Co_0.15_/N‐C catalyst suggested that MeOH overmatched EtOH, ^n^PrOH, ^i^PrOH, and THF (Figure S12, Supporting Information), where the total monomers yields, selectivity to **S1** and **G1,** and the delignification degrees approximately followed linear relationships with the solvent polarity (*E*_T_(30) values) (Figure 3H; Figure S13, Table S6, Supporting Information).^[^ ^36^ ^]^ These results suggested that a high polar solvent makes for extracting lignin fragments from the biomass matrix into solvent and the hydrogenation procedure.^[^ ^35^ ^]^ The influences of temperature, hydrogen pressure, and catalyst dosage were all investigated (Figure 3I–K; Figures S14–S16, Tables S7–S9, Supporting Information). When the reaction conditions were strengthened, the yields of monophenols and the selectivity to **S1** and **G1** were both increased, along with declining trends in propenyl‐substituted phenols **S2** and **G2**. Under the optimized conditions, that is 200 °C, 3 MPa H_2_, and 10 wt.% of catalyst, high yields of **S1** and **G1** could be achieved over Co_0.15_/N‐C catalyst in MeOH. These results indicated that monophenols with a propenyl end‐chain should be in the active intermediate state toward **S1** and **G1** during lignin hydrogenolysis.^[^ ^46^ ^]^

### 2.6 RCF of Various Lignocellulose

**Figure 4:**
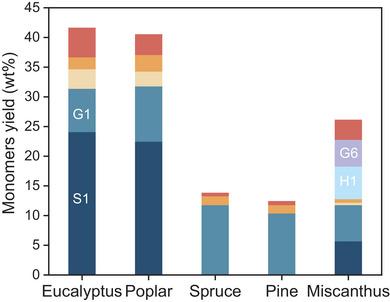
RCF of different lignocellulosic feedstocks. **H1** and **G6** represent methyl 3‐(4‐hydroxyphenyl) propanoate and methyl 3‐(4‐hydroxy‐3‐methoxyphenyl) propanoate, respectively.

Encouraged by these findings, the Co_0.15_/N‐C catalyst was applied to the RCF of other fast‐growing wood and grass biomass (**Figure** **4**). In the case of other hardwoods, such as eucalyptus and poplar, phenolic monomers were generated in near theoretical maximum yields (41.6 wt.% and 40.5 wt.%, respectively) with high selectivity to **S1** and **G1**. Given that lignin in softwoods (such as pine and spruce) is mainly composed of G‐units with less cleavable linkages, the RCF resulted in monomeric phenols in 12.4–13.8 wt.% yields, where **G1** was established as a dominant product. Miscanthus, an important upright energy grass, in which *p*‐hydroxybenzoates, such as *p*‐coumaric acid (*p*CA) and ferulic acid (FA) moieties, link on the sidechains of the lignin, was also treated with the Co_0.15_/N‐C catalyst. In this context, phenolic monomers including **S1** (5.7 wt.%) and **G1** (6.1 wt.%) from the cleavage of β‐O‐4 linkage, as well as **H1** (4.5 wt.%) and **G6** (5.5 wt.%) from *p*‐hydroxybenzoates, were formed in a 26.1 wt.% combined yield (Figure S17 and Table S10, Supporting Information).

### 2.7 Mechanistic Study

**Figure 5:**
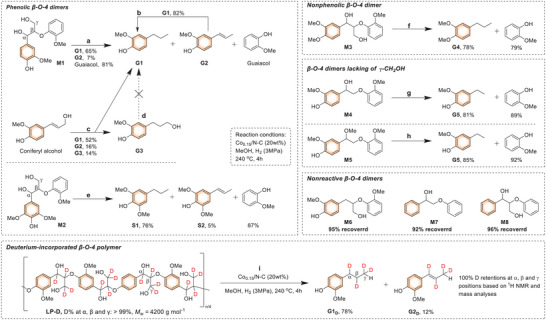
Reactivity of β‐O‐4 lignin mimics with Co_0.15_/N‐C. Reaction conditions: substrate (25 mg), Co_0.15_/N‐C (5 mg, 20 wt.%), MeOH (10 mL), 240 °C, H_2_ (3 MPa at 25 °C), and 4 h.

To elucidate the mechanism of lignin hydrogenolysis over the Co_0.15_/N‐C catalyst, the reactivities of various β‐O‐4 model compounds were screened (**Figure** **5**). A phenolic β‐O‐4 dimer **M1** that can mimic the terminal units of lignin biopolymer, gave **G1** as a major product (65%), along with the observation of **G2** (7%) (reaction a). This product distribution was kin to those from the RCF of lignocellulose. Catalytic treatment of **G2** resulted in **G1** efficiently, suggesting that **S1** may be an intermediate to **G1** during lignin depolymerization (reaction b). In the case of coniferyl alcohol as a substrate, in addition to **G1** and **G2**, a nonnegligible amount of **G3** (14%) was formed (reaction c). The scenarios that **G3** could not be converted into **G1** or **G2** (reaction d), and **G3** has not been observed in current lignocellulose RCF, hinted that coniferyl alcohol may be not enroute to the formation **G1**. In this context, it was deduced that the cleavage C_γ_‐OH may occur at the β‐O‐4 dimeric form. In the case of dimer compounds **M2** having a syringyl unit (reaction e), and **M3** mimicking internal units of lignin (reaction f), corresponding propyl‐chained arenes were smoothly generated through the cleavage of β‐O‐4 moieties (Figures S18 and S19, Supporting Information). Two imperfect lignin β‐O‐4 models **M4** and **M5** that lack the ‐CH_2_OH unit could be depolymerized into monomeric products in high yields, suggesting its trivial role during the cleavage of β‐O‐4 units (reactions g and h) (Figure S20, Supporting Information). On the contrary, β‐O‐4 model **M6** without α‐OH could not be depolymerized under such a condition, and this scenario was in line with the previous results.^[^ ^35^, ^46^ ^]^ Of note, dimer **M8** having an integral β‐O‐4 skeleton but lacking OH and/or MeO groups on arenes, displayed chemical inertness over the Co_0.15_/N‐C catalyst. The same happened on **M7** (Figure S21, Supporting Information). These results demonstrated that aromatic OH and MeO species, the indispensable units in lignin biopolymer, play a key role in lignin hydrogenolysis.

β‐O‐4 linkages contain a secondary benzylic alcohol and a primary aliphatic alcohol, which have displayed diversified paths during lignin depolymerization.^[^ ^46^ ^]^ For example, dehydration and/or dehydrogenation reactions where the native linkage protons would be partially lost, have been proposed in the hydrogenolysis mechanisms.^[^ ^46^ ^]^ To further clarify the path of lignin hydrogenolysis, a β‐O‐4 polymer with deuterium incorporated at the α, β, and γ positions was treated with Co_0.15_/N‐C (reaction i). D‐incorporated guaiacol derivatives **G1‐D** and **G2‐D** were formed in 78% and 12% yields, respectively. The analyses of the ^1^H NMR spectrum and mass data of isolated **G1‐D** indicated that the deuteriums in β‐O‐4 polymer were well preserved in the final product after hydrogenolysis, thus ruling out the possible routes containing the D‐loss steps (dehydration and/or dehydrogenation) (Figure S22, Supporting Information). To meet the requirements of both the propenyl‐substituted **G2** as an intermediate and the no participation of the β‐*O*‐4 linkage protons, we hypothesized that the C_α_–OH and C_β_–O bonds are simultaneously broken in β‐O‐4 moieties by a synergistic hydrogenolysis mechanism. This is consistent with the results we reported earlier.^[^ ^35^, ^46^ ^]^

### 2.8 Lignin Derivable Polyesters

The distinctive aromatic backbone of lignin makes it a potentially valuable feedstock for producing functional polymers with a low‐carbon footprint, and this approach could also add significant profitability to biorefinery.^[^ ^6^, ^14^, ^41^ ^]^ As the RCF develops, utilizing lignin‐depolymerized monomers (not model compounds or lignin‐mimics) to produce designer chemicals and materials has become a reality recently.^[^ ^3^, ^20^, ^28^, ^34^, ^41^, ^47^, ^48^, ^49^ ^]^ Because these methoxy‐ and alkyl‐adorned lignin monomers are seldom used in current chemical and material industries, the search for new routes for fabricating lignin‐derivable polymers is still a motivating aspiration.

**Figure 6:**
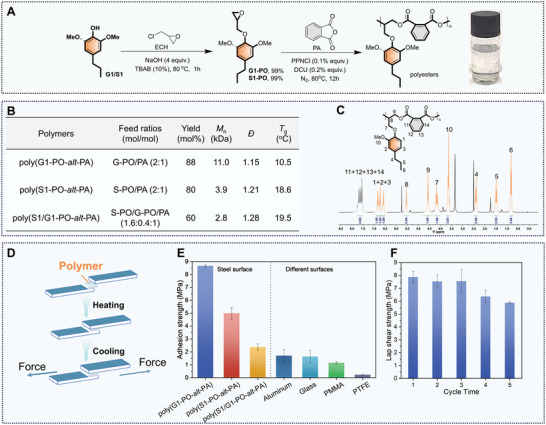
Synthesis and performance of polyesters. A) Synthesis route of polyesters. B) Characterization of polyesters. C) ^1^H‐NMR spectra of poly(G1‐PO‐*alt*‐PA). D) Schematic diagram of the adhesion and lap shear procedure. E) Comparison of lap shear strength of poly(G1‐PO‐*alt*‐PA), poly(S1‐PO‐*alt*‐PA), poly(S1/G1‐PO‐*alt*‐PA) on steel surfaces or different surfaces. F) Multiple recycle lap shear tests of poly(G1‐PO‐*alt*‐PA).

The ring‐opening alternating copolymerization (ROAC) of epoxides with cyclic anhydrides has become a prospective route to construct miscellaneous polyesters with various properties and functions.^[^ ^50^, ^51^, ^52^ ^]^ Thereby, it was chosen as a strategy to demonstrate the utility of lignin‐depolymerized monomers. As shown in **Figure** **6**A, the reaction of compound **G1** or **S1** with epichlorohydrin enabled the formation of corresponding epoxides in high yields. The combination of bis(triphenylphosphine)iminium chloride (PPNCl) and 1,3‐dicyclohexylurea (DCU) was selected as a catalyst for ROAC of **G1‐PO** with phthalic anhydride (PA) (Figure 6A). With the feed ratio of DCU/PPNCl/G1‐PO/PA = 1:2:1000:500, the copolymerization occurred smoothly at 80 °C under solvent‐free conditions, affording poly(G1‐PO‐*alt*‐PA) in 88% yield with a high molecular weight (*M*_n_ = 11 kDa), narrow molecular weight distribution (*Đ* = 1.1) (Figure 6B). This reaction showed >99% alternating degree based on the analyses of ^1^H NMR and ^13^C NMR spectra (Figure 6C; Figure S28, Supporting Information). ROAC of **S1‐PO** and PA gave a polymer poly(S1‐PO‐*alt*‐PA) in 80% yield with *M*_n_ = 3.9 KDa and *Đ* = 1.21 (Figure S23, Supporting Information). A mixture containing **S1** and **G1**, that was isolated from RCF of silver birch without further fractionation, could also undergo epoxidation and copolymerization, which gave poly(S1/G1‐PO‐*alt*‐PA) in 60% yield. The glass transition temperature (*T*_g_) of poly(G1‐PO‐*alt*‐PA) (10.5 °C) with one *ortho*‐MeO group was lower than poly(S1‐PO‐*alt*‐PA) (18.6 °C) and poly(S1/G1‐PO‐*alt*‐PA) (19.5 °C) (Figure 6B; Figure S24, Supporting Information), probably because two *ortho* methoxy groups constrained rotation of the pendant group.^[^ ^41^ ^]^ Thermogravimetric analysis (TGA) indicated that poly(G1‐PO‐*alt*‐PA) manifested excellent thermal stability (*T*_5%_ = 292 °C), being better than those having **S1‐PO** units (193 °C and 189 °C) (Figure S25, Supporting Information).

Despite many cases of ROAC, their functionality has yet to be exploited. Herein, the adhesion performance of resultant lignin‐derived polyesters was evaluated by the lap shear experiments. These polymers were first deposited on the surfaces of the substrates and then by gentle heating followed by pressure, with no tedious solidification processes (Figure 6D). Using steel as an example, the average adhesion strengths of poly(G1‐PO‐*alt*‐PA) were measured as 8.6 MPa under dry conditions, which was superior to the performance of most typical adhesives.^[^ ^53^, ^54^, ^55^, ^56^ ^]^ By comparison, poly(S1‐PO‐*alt*‐PA) and poly(S1/G1‐PO‐*alt*‐PA) displayed weaker adhesion effects (5.0, 2.3 MPa, respectively), probably due to their relatively low average molecular weights.^[^ ^57^ ^]^ Poly(G1‐PO‐*alt*‐PA) also showed excellent adhesion effects on other hydrophilic surfaces, including aluminum (1.70 MPa) and glass (1.63 MPa). The adhesion effect of poly(G1‐PO‐*alt*‐PA) on hydrophobic surfaces was relatively weak, as seen in the case of PMMA (1.15 MPa) and PTFE (0.233 MPa) (Figure 6E). This is because H‐bonding, the main adhesion force in most supramolecular adhesion systems, is unfavorable for adhesion onto hydrophobic surfaces. Of note, the adhesion strengthes of poly(G1‐PO‐*alt*‐PA) on PMMA and PTFE are still much higher than previously reported supramolecular adhesives.^[^ ^58^, ^59^, ^60^, ^61^ ^]^ The repeated peeling/adhering cycle experiments implied that the poly(G1‐PO‐*alt*‐PA) featured excellent reusable adhesiveness, where almost 68% of the maximum strength remained after 5 cycles (Figure 6F).

## 3 Conclusion

We designed an inexpensive, highly atomically dispersed, ultra‐low‐doped, cobalt‐based catalyst. It was employed for the RCF of lignocellulosic biomass, from which high yield and high selectivity of phenolic monomers, together with high retentions of carbohydrate, were realized. Current RCF treatments demonstrated the entire utilization of all lignocellulose components, 1) the lignin‐depolymerized monomers were isolated, derivatized, and polymerized to robust adhesives; 2) anti‐solvent precipitation of lignin‐derived dimers and oligomers generated lignin nanoparticles; 3) highly retained carbohydrate pulp was efficiently enzymolyzed into sugars. The reactivity study of the lignin model compounds illustrated the synergistic hydrogenolysis of C_α_–O and C_β_–O mechanism. This study developed a cost‐effective catalyst for lignin valorization and the entire utilization of all components of biomass.

## 4 Experimental Section

### Materials

Silver birch wood (*Betula pendula* Roth), eucalyptus grandis (*Eucalyptus*), poplar wood (*Populus tomentos*), spruce wood (*Picea*), and pine wood (*Pinus*) were crushed, screened into 40–60 meshes, extracted with toluene/ethanol and dried at 65 °C for at least 24 h before use. Cobalt nitrate hexahydrate was purchased from Aladdin. Gallic acid, melamine, methanol, ethanol, isopropanol, propanol, tetrahydrofuran, bis(triphenylphosphine)iminium chloride (PPNCl), 1,3‐dicyclohexylurea (DCU), phthalic anhydride (PA), tetrabutylammonium bromide (TBAB), Epichlorohydrin (ECH) were supplied by Energy Chemical; Tetradecane, activated carbon (AC) were purchased from Sigma Aldrich; Deionized water was supplied by Guangzhou Watsons Food & Beverage Co. Ltd. All commercial reagents in this study were used as received, unless otherwise noted.

### Preparation of Co_0.15_/N‐C Catalyst

Cobalt nitrate hexahydrate (80 mg) and gallic acid (1.09 g) were dissolved in ethanol. Subsequently, activated carbon (1.5 g) was introduced into the mixed solution, resulting in a black homogeneous suspension achieved through ultrasound treatment for 0.5 h. The solid residue was obtained by repetitive washing and centrifugation in an ethanol solution, followed by drying at 60 °C for 24 h to yield a dry solid powder. This powder was then combined with melamine (1.5 times the mass of the solid powder) and ground for 1 h to form the catalyst precursor. The precursor was meticulously loaded into a quartz boat and subjected to calcination at 800 °C for 2 h under nitrogen atmosphere. Consequently, the Co_0.15_/N‐C catalyst was successfully obtained.

### Catalytic Hydrogenolysis of Lignocellulose over Co_0.15_/N‐C

In a typical experimental procedure, 250 mg of silver birch wood chips (60–80 mesh), Co_0.15_/N‐C catalyst (50 mg), and 15 ml of methanol were introduced into a 50 ml Parr reactor. Subsequently, the reactor was sealed, and nitrogen was purged through it four or five times to eliminate any residual air before injecting hydrogen to achieve a pressure of 3 MPa inside the reactor. The temperature of the reactor was gradually raised to the desired temperature (240 °C) over 1.5 h, and the reaction was conducted at this temperature for a duration of 4 h. The reactor pressure could reach 12.0 MPa by the end of the reaction. Upon completion, the reactor was cooled to room temperature, and solid–liquid separation was performed using a 0.22 µm filter. The solid residue obtained was a mixture of catalyst and carbohydrate, while the liquid phase contained lignin oil. After removing methanol by rotary evaporation, the resulting oil was extracted using CH_2_Cl_2_, and subsequent analysis was conducted using GC‐MS and GC to identify and quantify the components.

### Synthesis of G1‐PO

The monomer **G1** (5.0 g, 30.1 mmol), ECH (6.97 g, 75.3 mmol), and TBAB (0.97 g, 3.01 mmol) were mixed in a round‐bottomed flask and refluxed at 80 °C for 1 h. Subsequently, a mixture of 20 wt.% sodium hydroxide (0.48 g, 120.4 mmol) and TBAB (0.97 g, 3.01 mmol) was dropwise added to the flask under ice bath conditions, followed by a reaction at 30 °C for 0.5 h. The resulting **G1‐PO** monomers were obtained through simple extraction, water removal, rotary evaporation and silica gel column chromatography.

### Synthesis of Polyesters

**G1‐PO** or **S1‐PO** (1.0 g, 4.5 mmol), PA (0.333 g, 2.25 mmol), PPNCl (2.58 mg, 0.0045 mmol), and DCU (2.0 mg, 0.009 mmol) were mixed in a 25 mL Schlenk flask. The reaction proceeded at 80 °C under a nitrogen atmosphere for 12 h. After cooling to room temperature, the mixture was dissolved in dichloromethane, precipitated into excess n‐hexane, and subjected to multiple dissolution and precipitation cycles to remove unreacted monomers. The final polyesters were obtained after rotary evaporation and vacuum drying.

## Conflict of Interest

The authors declare no conflict of interest.

## Author Contributions

G.S. and X.L., conceived the project. X.L. designed and synthesized the catalysts and performed the catalysts characterizations and catalytic experiments. X.L., S.W., and H.L. carried out the catalytic hydrogenolysis of lignin model compounds. X.G. assisted in XAFS data analyses. R.M. performed the hydrothermally treated catalysts. G.S. and X.L. wrote the manuscript. All authors discussed the results and commented on the manuscript.

## Supporting information

Supporting Information

## Data Availability

The data that support the findings of this study are available in the supplementary material of this article.
