# Supplementary material for: Harnessing Atomically Dispersed Cobalt for the Reductive Catalytic Fractionation of Lignocellulose
Source: Adv Sci (Weinh). 2024 Mar 17;11(22):2310202. doi: 10.1002/advs.202310202 (PMC11165530; doi:10.1002/advs.202310202)
Supplement: Supplementary file 1 — Supporting Information [file ADVS-11-2310202-s001.pdf]

ADVANCED SCIENCE
Open Access

Supporting Information

for *Adv. Sci.*, DOI 10.1002/advs.202310202

Harnessing Atomically Dispersed Cobalt for the Reductive Catalytic Fractionation of Lignocellulose

*Xiancheng Li, Rumin Ma, Xueying Gao, Helong Li, Shuizhong Wang and Guoyong Song**

ADVANCED SCIENCE
Open Access

Supporting Information

Harnessing Atomically Dispersed Cobalt for the Reductive Catalytic Fractionation of Lignocellulose

*Xiancheng Li, Rumin Ma, Xueying Gao, Helong Li, Shuizhong Wang, Guoyong Song**

Supplementary Text

GPC analysis

GPC analyses were performed on Shimadzu LC-20AD equipped with a PL-gel 3 μm Mixed-E column (mixed) and UV detection detector (254 nm) at 50 ^{o}C. THF was used as the mobile phase with a 1mL min^{-1} flow rate. 1) GPC samples were prepared by dissolving 2 mg of lignin oil in 1 mL of THF and filtered with a 0.22 μm PTFE filter prior to injection. Six GPC polystyrene standards (162-1860 g mol^{-1}) were used for calibration. 2) The as-obtained polymers were dissolved in THF to form solutions (2 mg mL^{-1}), which were then filtered with a 0.22 μm filter to remove any particle matter. The sample injection volume was 20 uL, the flow rate was 1 mL min^{-1}, and the column temperature was 50 ^{o}C. The system was calibrated using polystyrene standards with a molecular weight range of 1860-37900 g mol^{-1}.

GC and GC-MS analysis

GC and GC-MS analyses were carried out on Shimadzu GC 2010 series equipped with a HP-5 column using a flame ionization detector (FID) and a Shimadzu GC 2010 series equipped with a HP-5 MS column, respectively. The following GC or GC-MS procedure was used: 1 μL aliquot with a split ratio of 20:1, injection temperature of 250 ^{o}C, column temperature program: 50 ^{o}C (hold time 3 min), 8 ^{o}C min^{-1} to 280 ^{o}C (hold time 5 min), detection temperature of 290 °C (for FID) or 280 ^{o}C (for MS). The quantification of monomers in the lignin oily samples were evaluated with authentic samples using commercially available or independently synthesized standards. The as-obtained lignin oils were diluted to 10 mg mL^{-1} in ethyl acetate containing internal standard (tetradecane) before GC and GC-MS systems. Linear calibration curves were produced from 0.14 mg mL^{-1} to 3.9 mg mL^{-1} for authentic samples.

The lignin oil yields were calculated based on the Klason lignin weight, calculating formula as shown below:

$$\text{Phenolic monomers (wt\%)} = \frac{\text{Mass (total monomers)}}{\text{Mass (Klason lignin)}} \times 100\% \quad (1)$$

$$\text{Delignification (wt\%)} = \frac{\text{Mass (Klason lignin)-Mass (lignin in pulp)}}{\text{Mass (Klason lignin)}} \times 100\% \quad (2)$$

$$\text{Sugar retention (wt\%)} = \frac{\text{Mass (Sugar in pulp)}}{\text{Mass (Sugar in sawdust)}} \times 100\% \quad (3)$$

HPLC analysis

HPLC analysis was conducted on a Shimadzu LC-20A equipped with an aminex HPX-87H column (300 × 7.8 mm) with 9 μm particle size using a differential refractive index detector (RID-20A). The detection temperature was 60 °C, the velocity of the pump was 0.6 mL min^{-1} (5 mM H_2SO_4).

NMR analysis

The NMR spectra were acquired on a Bruker Avance III HD 500MHz spectrometer instrument (Bruker, Germany). Lignin, oily products and synthetic lignin polymers were dissolved in DMSO-d_6, whereas lignin models were dissolved in $CDCl_3$. As for 2D HSQC NMR experiment, the solvent peak (DMSO-d_6) at δ_C/δ_H 39.5/2.49 ppm was used as an internal reference.

XRD analysis

X-ray diffraction (XRD) analysis was carried out using a Rigaku SmartLab SE diffractometer with a Cu Kα radiation source operated at 40 mA and 40 kV. XRD patterns were collected in the 2θ range of 5° to 90° at a scan speed of 2° min^{-1}.

XPS analysis

X-ray photoelectron spectroscopy (XPS) analysis was conducted using a Thermo Scientific K-Alpha spectrometer with a monochromatic Al K_α X-ray source operated at a voltage of 1486.6 eV. All spectra were collected with a step of 0.1 eV and a pass energy of 50 eV using the C 1s binding energy of 284.8 eV for calibration.

HR-TEM analysis

High resolution transmission electron microscopy (HR-TEM) images were taken with JEOL JEM-F200 microscope operated at an acceleration voltage of 200 kV.

BET analysis

The Brunauer-Emmett-Teller (BET) surface area was calculated from a multipoint BET analysis of the nitrogen adsorption isotherms and the N_2 adsorption–desorption isotherm was performed with Builder SSA-7000 apparatus at 77K.

ICP-OES analysis

Inductively Coupled Plasma–Optical Emission Spectrometry (ICP–OES) was performed on ICP-OES 730 (Agilent) to determine the metal elemental of catalysts.

HAADF-STEM analysis

High-angle annular dark-field scanning transmission electron microscopy (HAADF-STEM) images and corresponding elemental mapping were carried out on JEM-ARM300F with a spherical aberration corrector at 300 kV.

The X-ray absorption fine structure (XAFS) analysis

The XAFS measurements of $Co_{0.15}$/N-C were performed at Beijing Synchrotron Radiation Facility (BSRF). The acquired EXAFS data were processed according to the standard procedures using the Athena and Artemis implemented in the IFEFFIT software packages. The fitting detail is described below according to the reported literature.[1] The EXAFS spectra were obtained by subtracting the post-edge background from the overall absorption and then normalizing with respect to the edge-jump step. Subsequently, the χ(k) data of Co K-edge were Fourier transformed to real (R) space using a Hanning window (dk = 1.0 $Å^{-1}$) to separate the EXAFS contributions from different coordination shells. To obtain the quantitative structural parameters around central atoms, least-squares curve parameter fitting was performed using the ARTEMIS module of IFEFFIT software package.[2] The following EXAFS equation was used.

$$\chi(k)=\sum_j \frac{N_j S_o^2 F_j(k)}{k R_j^2}\exp[-2k^2\sigma_j^2]\exp[\frac{-2R_j}{\lambda(k)}]\sin[2kR_j+\phi_j(k)]$$

S_0^2 is the amplitude reduction factor, $F_j(k)$ is the effective curved-wave backscattering amplitude, N_j is the number of neighbors in the jth atomic shell, R_j is the distance between the X-ray absorbing central atom and the atoms in the jth atomic shell (back scatterer), λ is the mean free path in Å, $\phi_j(k)$ is the phase shift (including the phase shift for each shell and the total central atom phase shift), σ_j is the Debye-Waller parameter of the jth atomic shell (variation of distances around the average R_j). The functions $F_j(k)$, λ, and $\phi_j(k)$ were calculated with the ab initio code FEFF8.2. The coordination numbers of model samples were fixed as the nominal values. The obtained S_0^2 was fixed in the subsequent fitting. While the internal atomic distances R, Debye-Waller factor σ^2, and the edge-

energy shift ΔE_0 were allowed to run freely.

Atomic force microscopy analysis (AFM)

The atomic force analysis was conducted with a Bruker Multimode 8 (USA). The samples were prepared by applying suspensions of LNPs (0.001 mg mL^{-1}) onto fresh mica substrates. The tapping mode Atomic Force Microscopy (AFM) imaging was obtained under conditions with a scanning range of 5 μm and a scanning speed of 1 Hz.

Thermogravimetric analysis (TGA)

TGA was conducted on TG 209 F3 Tarsus (Netzsch, Germany). The detection temperature ranged from 30 to 600 °C with a heating rate of 10 °C min^{-1} under a nitrogen flow of 10 mL min^{-1}.

Differential scanning calorimetry analysis (DSC) analysis

Differential scanning calorimetry (DSC) analysis was performed using a TG 209 F3 Tarsus instrument (Netzsch, Germany) under a nitrogen atmosphere. The sample (5-10 mg) underwent a thermal treatment protocol, initially ramping from room temperature to 200 °C to eliminate any thermal history, followed by a decrease from 200 to -50 °C, and a subsequent increase to 150 °C. Both the cooling and heating rates were set at 10 °C min^{-1}.

Lap Shear Adhesion Test

The adhesive strength of the polyester was assessed using an electronic universal testing machine (CMT6103). A 5 mg quantity of polyester, dissolved in dichloromethane, was uniformly applied to the substrate with a coating area of 10 × 10 mm^2. The coated samples were subjected to a vacuum oven at 80°C for 0.5 hours to eliminate dichloromethane. Subsequently, the polyester-coated substrates were overlapped and securely fastened with dovetail clamps before being placed in an oven at 100 °C for 2 hours. Following cooling to room temperature, the specimens were stored for 12 hours before undergoing mechanical testing.

Table S1. ICP-OES analysis for the $Co_{0.15}$/N-C catalysts

Catalyst	ICP-OES analysis (wt%)	S_{BET}	V_{pore}	D_{pore}
	Co	(m^2 g^{-1}) [a]	(cm^3 g^{-1}) [b]	(nm) [c]
Fresh $Co_{0.15}$/N-C	0.15	793	0.63	1.88
Spent $Co_{0.15}$/N-C	0.14	198	0.30	1.92
Activated $Co_{0.15}$/N-C [d]	0.10	424	0.40	1.93
AC	/	830	0.54	1.91

[a] BET surface area.

[b] BJH desorption cumulative volume of pores.

[c] BJH desorption average pore diameter.

[d] Activated $Co_{0.15}$/N-C represents it was regenerated under 600°C for 2 h.

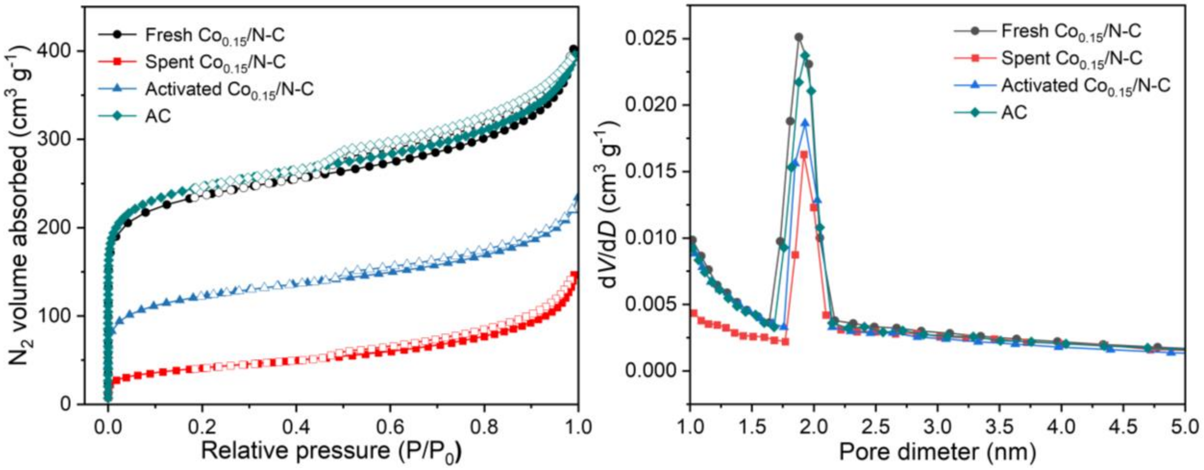

Figure S1. BET analyses of fresh, spent, activated $Co_{0.15}$/N-C catalysts and activated carbon. (a) N_2 adsorption-desorption isotherms, and (b) pore dimeter distribution plots for fresh, spent, activated $Co_{0.15}$/N-C catalysts and activated carbon.

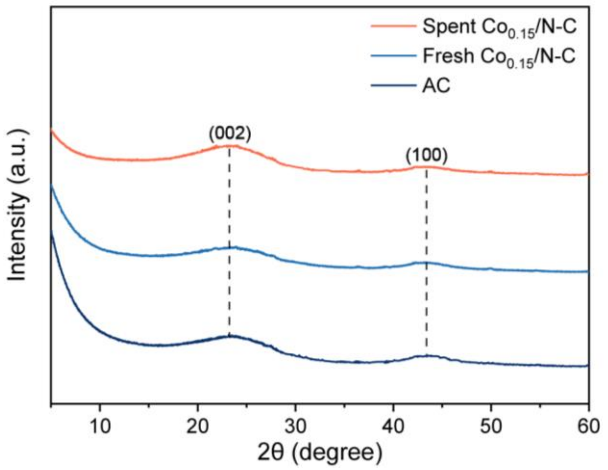

Figure S2. XRD pattern of fresh, spent $Co_{0.15}$/N-C catalyst and activated carbon.

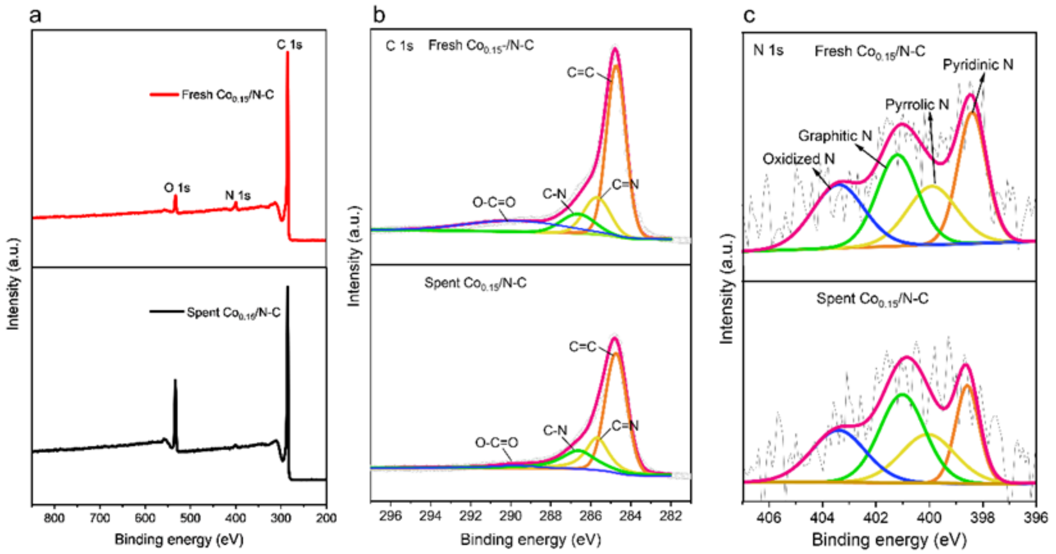

Figure S3. XPS analysis of fresh, spent $Co_{0.15}$/N-C catalysts. (a) XPS spectra, high-resolution spectra of (b) C 1s, (c) N 1s.

Table S2. EXAFS fitting parameters at the Co K-edge for various samples (S_0^2=0.74 from Co-foil)

Samples	Shell	CN[a]	R[b](Å)	σ^{2c}(Å^2)	ΔE_0^d(eV)	R factor
Co-foil	Co-Co	12	2.49±0.01	0.0064	7.2±0.3	0.0011
CoO	Co-O	6	2.12±0.02	0.0125	-3.1±0.7	0.0066
	Co-Co	12	3.00±0.01	0.0088	-3.1±0.7	
CoPc	Co-N	4	1.91±0.01	0.0023	6.4±2.4	0.0117
$Co_{0.15}$/N-C	Co-N	4.1±0.2	1.96±0.01	0.0094	-7.3±1.8	0.0039

[a]*CN*: coordination numbers; [b]*R*: bond distance; [c]σ^2: Debye-Waller factors; [d] ΔE_0: the inner potential correction. R factor: goodness of fit. Error bounds that characterize the structural parameters obtained by EXAFS spectroscopy were estimated as CN±20%; R ±1%; σ^2 ±20%.

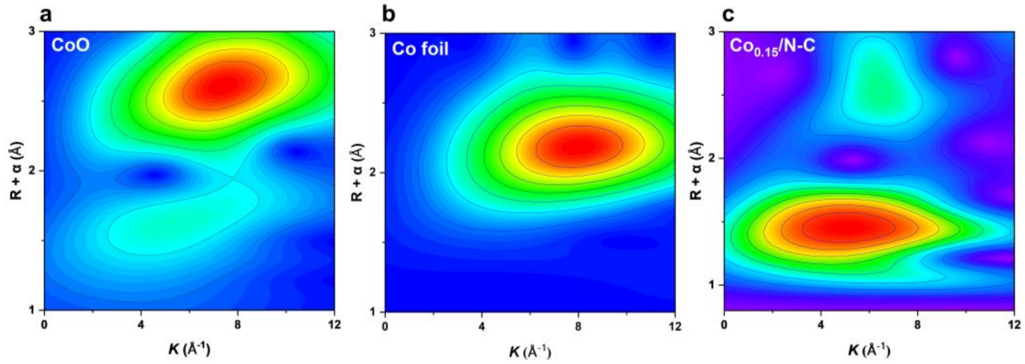

Figure S4. Wavelet transformed (WT) k^2-weighted $\chi(k)$-function of the Ru-K edge EXAFS spectra. (a) CoO, (b) Co foil, and (c) $Co_{0.15}$/N-C.

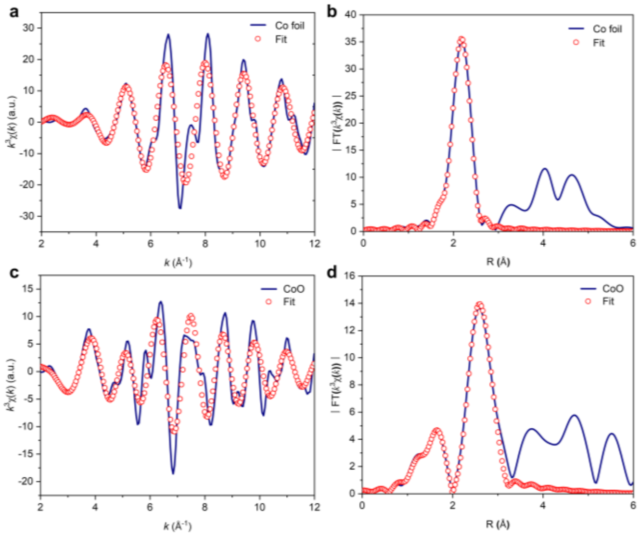

Figure S5. XAFS fitting curves. (a) k space EXAFS of Co foil, (b) FT-EXAFS of Co foil, (c) k space EXAFS of CoO, (d) FT-EXAFS of CoO.

Biomass compositional analysis

The composition of the biomass was analyzed according to the standard analytical procedures of the National Renewable Energy Laboratory (NREL). The general procedure was as follows: first the feedstock was extracted with toluene/ethanol (2:1, v/v) in a Soxhlet extractor for 12 hours and then dried at 105 °C for 8 hours. Lignocellulose (300 mg) was placed in a hydrolysis flask and ground with 72 wt% sulfuric acid solution (3.0 mL) for 1 h at room temperature followed by deionized water (84.0 mL). The hydrolysis flasks were then sealed and heated in an autoclave for 1 h at 120 °C. After cooling, the mixture was filtered through a mixed cellulose ester (MCE) membrane filter (0.2 μm). The precipitate was washed with water and dried at 105 °C and weighed to determine the acid insoluble lignin (AIL) content. The concentration of acid soluble lignin (ASL) was determined by UV spectroscopy to determine the absorbance at 205 nm of the soluble fraction of the filtrate. Determination of monomeric sugars in the filtrate was obtained by analysis on a high-performance liquid chromatography (HPLC) system equipped with an aminex HPX-87H column (300 × 7.8 mm) using a differential refractive index detector (RID-20A). These samples were analyzed in triplicate.

Table S3. Compositional analysis of various lignocellulosic biomass.[a]

Raw sawdust	AIL [b] (wt%)	ASL [c] (wt%)	Cellulose (wt%)	Hemicellulose (wt%)	Extraction (wt%)
Silver Birch	22.8	2.5	42.3	18.1	3.7
Eucalyptus	28.3	3.7	41.9	12.8	3.2
Poplar	23.7	2.8	46.9	16.0	3.3
Miscanthus	17.6	2.5	43.8	20.8	2.3
Spruce	32.9	1.4	43.6	14.4	2.2
Pine	30.1	0.8	42.5	9.3	4.3

[a] The compositions of biomass were analyzed according to the procedures of the NREL method.

[b] AIL: acid insoluble lignin (Klason lignin).

[c] ASL: acid soluble lignin.

Isolation of silver birch EMAL

Generally, the extracted silver birch powder was milled using a planetary ball-mill for 5 hours. The ball-milled birch powder (10 g) was treated with cellulase (5 mL) and xylanase (1 mL) in 200 mL citrate buffer (50 mM, pH 4.8) in an orbital water bath shaker at 50 °C for 48 h. After the enzymatic hydrolysis, the insoluble residue was collected by centrifugation, which was treated with cellulase and hemicellulase again under same conditions. The insoluble solid was collected, washed with HCl aqueous solution (pH = 2), and freeze-dried to afford crude lignin (5 g). The suspension of crude lignin in an acidic ([HCl] = 0.01 mol/L) mixture of dioxane/water (85:15 v/v, 100 mL) was refluxed under nitrogen for 4 h. The solid was collected by centrifugation and washed with dioxane/water (85:15, v/v). The combined supernatant was neutralized with sodium bicarbonate, followed by evaporation to afford a thick solution. This solution was added dropwise to a 1 L of HCl aqueous solution (pH = 2.0) to form a precipitate, which was allowed to equilibrate at 4 ºC overnight. The EMAL could be obtained by centrifugation, washing with HCl aqueous solution (pH = 2.0), and freeze-drying (2 g).

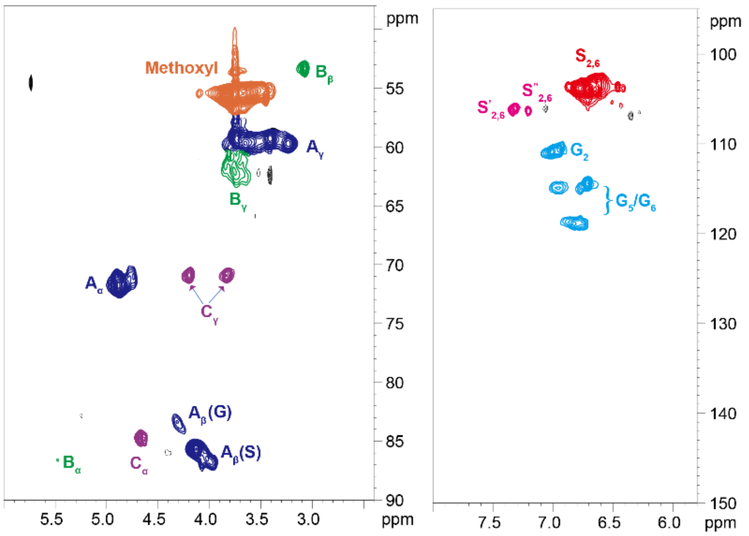

Figure S6. 2D HSQC NMR spectra of silver birch EMAL.

Table S4. Products distribution of catalytic hydrogenolysis of silver birch with different catalysts [a].

Catalyst	Monomers yield (wt%) [b]						Selectivity (mol%)	S/G	Delignification (wt%) [c]	M_w (g mol^{-1})	Sugar retention (wt%)	
	**G1**	**G2**	**S1**	**S2**	Others	Total	**G1+S1**				C5 retention	C6 retention
None	0.4	1.0	0.5	4.8	1.1	7.8	12	3.8	44	766	82	92
$Co_{0.15}$/C	0.8	2.9	2.4	11.8	2.2	19.9	16	3.5	61	415	85	89
N/C	0.6	0.9	0.6	5	1.0	8.1	15	3.7	49	631	81	87
$Co_{0.15}$/N-C	9.0	0.7	31.8	1.2	5.6	48.3	84	3.4	94	323	83	91
Ru/C	7.9	0.5	32.5	4.0	4.0	48.9	83	4.3	95	417	65	91
$Co_{0.15}$/N-C(H.T.)[d]	7.8	0.9	30.0	1.7	4.1	45.1	84	3.6	88	382	80	90

[a] Reaction conditions: birch sawdust (250 mg), catalyst (50 mg), H_2 (3 MPa), 240 °C, 4 h, MeOH (10 mL).

[b] Based on the Klason lignin content of birch sawdust

[c] Based on the weight of dichloromethane extracted fraction (Klason lignin)

[d] $Co_{0.15}$/N-C(H.T.) was treated by a hydrothermal (H.T.) process at 200 °C for 72 h.

To identify the probable dimers, the lignin oils (*ca.* 50 mg) were dissolved in anhydrous tetrahydrofuran (5 mL), to which *N*,*O*-bis(trimethylsilyl)trifluoroacetamide (BSTFA) (250 μL) was added. The mixture was stirred at 65 °C for 1 h under N_2, which was then analyzed on GC-MS. The following GC-MS procedure was used: 1 μL aliquot with a split ratio of 50:1, injection temperature of 250 °C, column temperature program: 50 °C (hold time 3 min), 5 °C min^{-1} to 280 °C (hold time 5 min), detection temperature of 280 °C. The probable structures of dimers were identified based on mass spectra analyses and reported literatures (*31*).

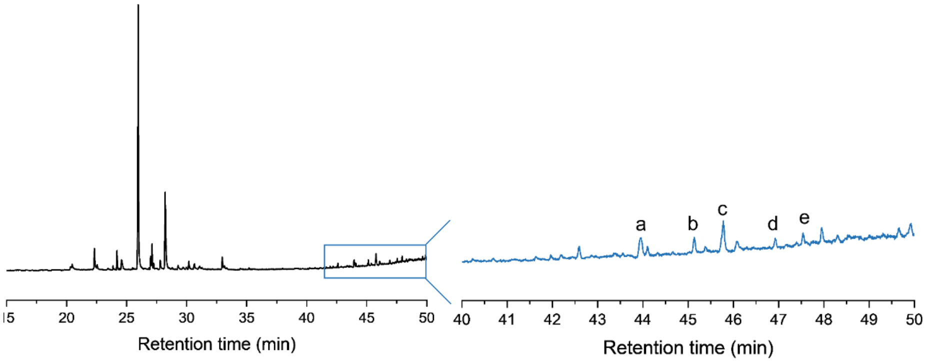

Dimer **a** Retention time: 43.959

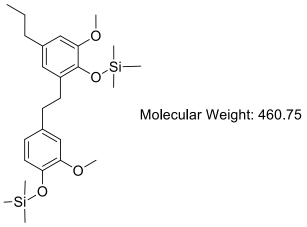

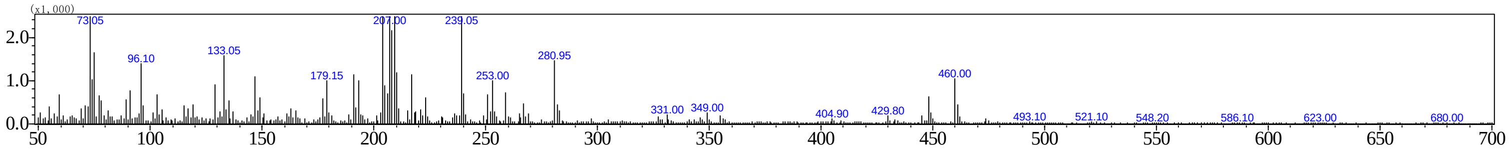

Dimer **b** Retention time: 45.135

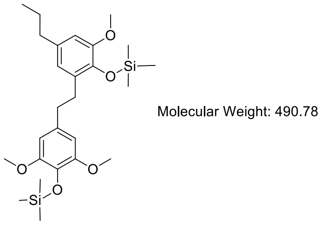

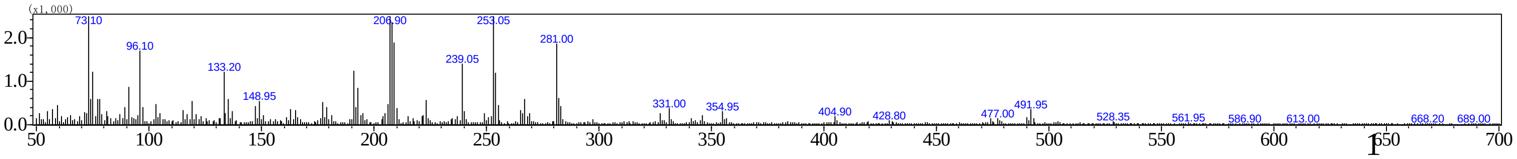

Dimer **c** Retention time: 45.779

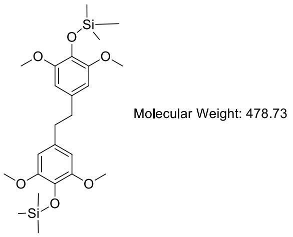

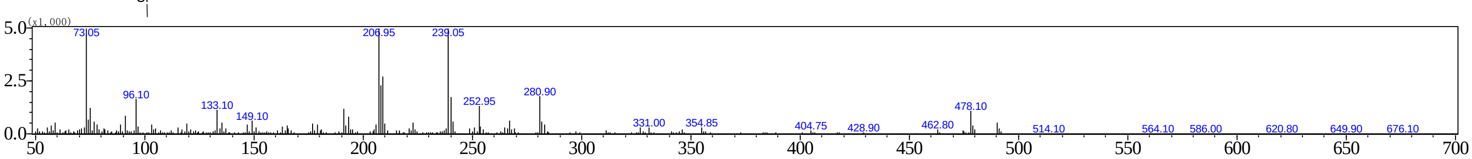

Dimer **d** Retention time: 46.692

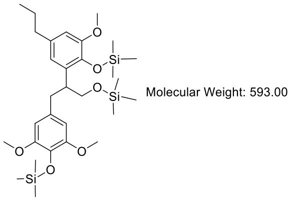

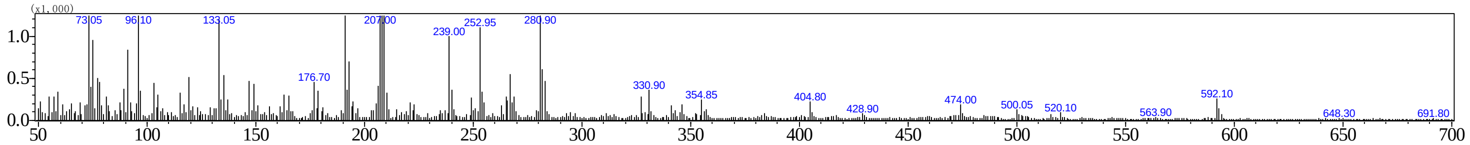

Dimer **e** Retention time: 47.541

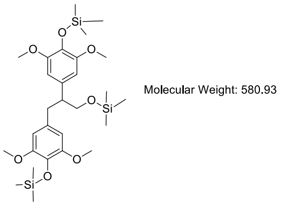

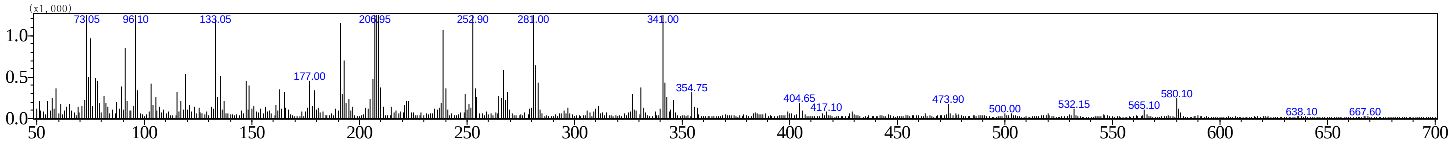

Figure S7. The information on the possible dimers of the lignin oils. GC-MS spectra of silylated lignin oils and structure analyses for possible dimers.

Synthesis of LNPs

The crude lignin oil (5.0 g) was extracted using a mixture of dichloromethane (DCM) and water. The soluble sugar products were separated to obtain purified lignin oil (4.5 g), and subsequently, lignin monomers, dimers and oligomers were further extracted using n-hexane to obtain a concentrated portion containing lignin-derived oligomers and dimers (1.8 g). The concentrated mixture (oligomers and dimers) was dissolved in tetrahydrofuran, and the solution was slowly added dropwise to a rapidly stirring beaker filled with distilled water, resulting in the formation of brown agglomerates. The water was then filtered off, and the LNPs (1.26 g) were finally obtained through freeze-drying.

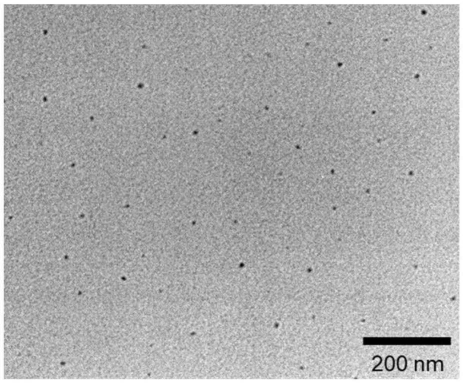

Figure S8. TEM images of LNPs.

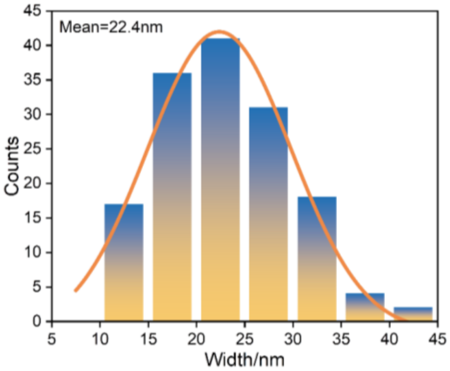

Figure S9. The width distribution of LNPs from AFM images.

Enzymatic treatment of carbohydrate pulp

500 mg of Carbohydrate residue, 20 mL of citric acid buffer solution (pH = 4.8, 50 mM), and 1mL of cellulase (Cellic@ CTec3, 100 FPU/mL) were mixed in a sealed conical flask and subjected to enzymatic hydrolysis in a thermostatic shaker at a constant temperature of 50 °C, the shaking rate of the shaker was 150 rpm. 100 μL of enzyme hydrolysis solution was taken at intervals of 1.5 hours from the beginning of the reaction, the solution was passed through the enzyme deactivation, and filtered to obtain the sample. It was further analyzed using HPLC. As a control birch sawdust was also treated in the same method.

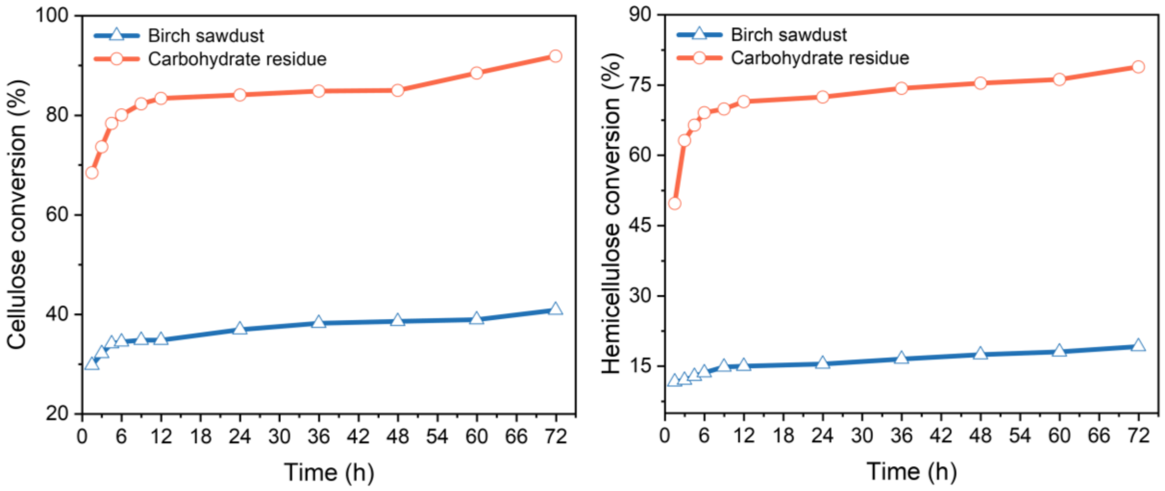

Figure S10. The enzymatic hydrolysis results of silver birch and carbohydrate residue.

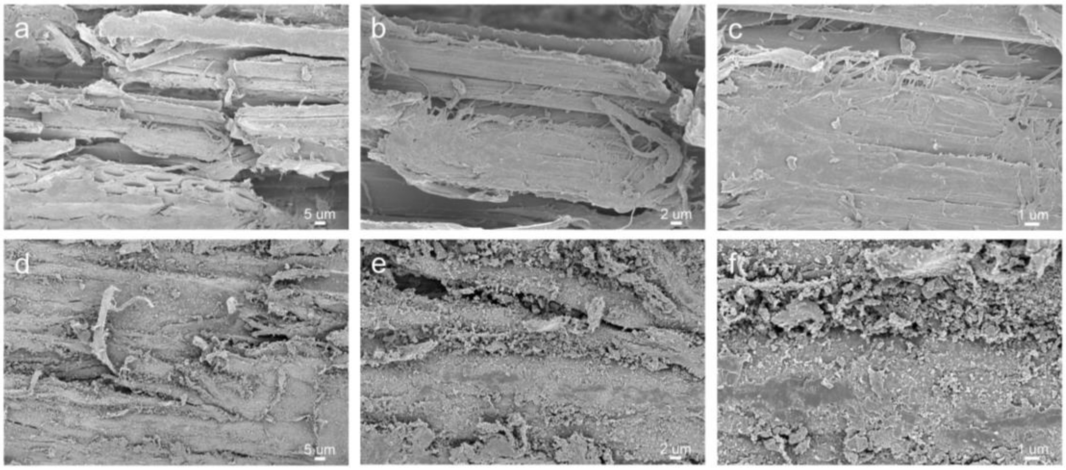

Figure S11. SEM images of different samples. (a-c) silver birch, (d-f) carbohydrate residue. Reaction conditions: silver birch (250 mg), $Co_{0.15}$/N-C (20 wt%), MeOH (15 mL), H_2 (3 MPa at 25 °C), 240 °C, and 4 h.

Table S5. Reusability of $Co_{0.15}$/N-C catalyst. [a]

Run time	Monomers yield (wt%) [b]						Selectivity (mol%)
	**G1**	**G2**	**S1**	**S2**	Others	Total	**G1+S1**
1st	9.0	0.7	31.8	1.2	5.6	48.3	84
2nd	4.3	2.3	18.5	9.1	7.3	41.5	55
3rd	2.6	2.8	16.5	10.0	5.6	37.5	51
4th	7.3	0.9	26	3.3	6.7	44.2	75
5th	7.0	0.7	25	6.9	2.6	42.2	76

[a] Reaction conditions: birch sawdust (250 mg), catalyst (50 mg), H_2 (3 MPa), 240 °C, 4 h, MeOH (10 mL).

[b] Based on the Klason lignin content of birch sawdust

1st refers to the fresh catalyst. For the 2nd, 3rd runs, the recovered catalyst was directly used after simple washing and drying. For the 4th, 5th runs, the recovered catalysts were calcinated at 600 °C for 2 h under N_2 flow.

Note: Separation the catalyst from carbohydrates. After RCF reaction, the solid phase containing carbohydrate pulp and catalyst was dried at room temperature, which was then transferred to a 100-mesh screening. The spent catalyst could be readily separated from the carbohydrate pulp by sieving, because the carbohydrate remained the original framework of biomass (2-5 mm) without collapse.

Table S6. Products distribution of $Co_{0.15}$/N-C-catalyzed RCF reaction of silver birch chips with different solvents. [a]

Solvent	Monomers yield (wt%) [b]						Selectivity (mol%)	Delignification (wt%) [c]	M_w (g mol^{-1})
	**G1**	**G2**	**S1**	**S2**	Others	Total	**G1+S1**		
MeOH	9.0	0.7	31.8	1.2	5.6	48.3	84	94	323
EtOH	7.3	0.8	25.4	1.6	3.8	38.9	84	82	344
^{i}PrOH	5.5	1.0	16.8	3.5	2.1	28.9	77	66	391
^{n}PrOH	7.6	0.7	21.3	1.8	4.0	35.4	82	73	368
THF	3.5	2.1	6.5	5.3	1.7	19.1	52	54	415

[a] Reaction conditions: birch sawdust (250 mg), catalyst (50 mg), H_2 (3 MPa), 240 °C, 4 h, solvent (10 mL).

[b] Based on the Klason lignin content of birch sawdust

[c] Based on the weight of dichloromethane extracted fraction (Klason lignin)

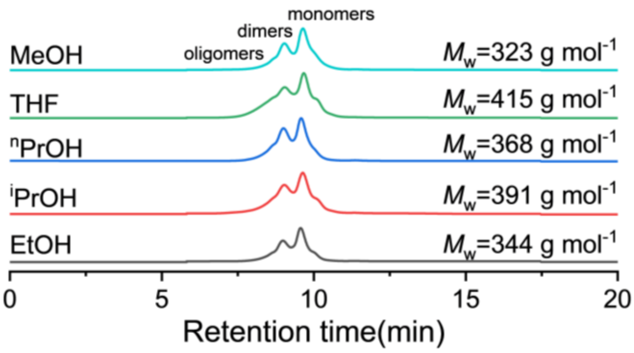

Figure S12. GPC spectra of lignin-derived products in different solvents. Reaction conditions: birch wood (250 mg), $Co_{0.15}$/N-C (50mg), 240 °C, H_2 (3MPa at 25 °C), and 4 h, solvent (10 mL).

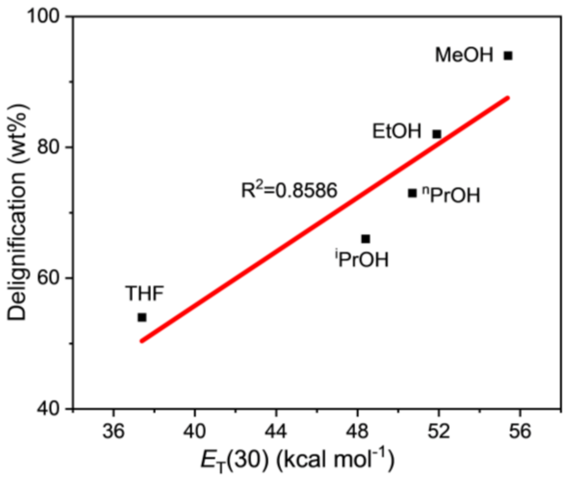

Figure S13. The relationship between the degrees of delignification and solvent polarity (E_T (30)).

Table S7. Products distribution of $Co_{0.15}$/N-C-catalyzed RCF reaction of birch at different reaction temperatures. [a]

Temperature (°C)	Monomers yield (wt%) [b]						Selectivity (mol%)	Delignification (wt%) [c]	M_w (g mol^{-1})
	**G1**	**G2**	**S1**	**S2**	Others	Total	**G1+S1**		
180	1.7	1.8	5.6	10.0	2.1	21.2	35	46	483
200	2.0	1.8	8.8	11.7	2.7	27.0	40	58	418
220	4.3	1.4	20.1	8.2	3.0	37.0	66	71	341
240	9.0	0.7	31.8	1.2	5.6	48.3	84	94	323
260	9.2	0.8	32.0	1.0	5.9	48.9	85	96	302

[a] Reaction conditions: birch sawdust (250 mg), catalyst (50 mg), H_2 (3 MPa), 4 h, MeOH (10 mL).

[b] Based on the Klason lignin content of birch sawdust.

[c] Based on the weight of dichloromethane extracted fraction (Klason lignin).

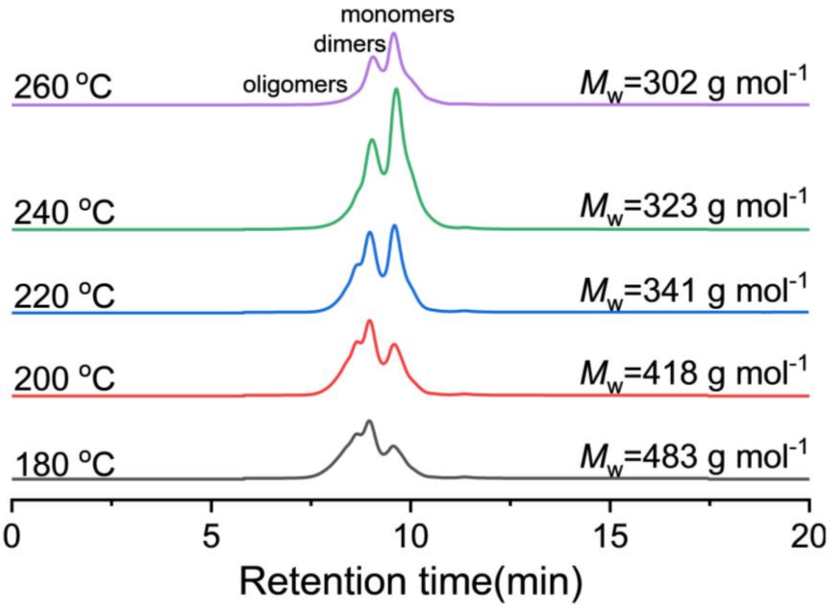

Figure S14. GPC spectra of lignin-derived products at different temperatures. Reaction conditions: birch wood (250 mg), $Co_{0.15}$/N-C (50mg), MeOH (10 mL), H_2 (3 MPa at 25 °C), and 4 h.

Table S8. Products distribution of $Co_{0.15}$/N-C-catalyzed RCF reaction of birch under different H_2/N_2 pressure . [a]

Pressure (MPa)	Monomers yield (wt%) [b]						Selectivity (mol%)	Delignification (wt%) [c]	M_w (g mol^{-1})
	**G1**	**G2**	**S1**	**S2**	Others	Total	**G1+S1**		
3[d]	1.1	1.9	1.8	7.1	0.9	12.8	23	56	582
1atm	ND	1.4	ND	2.9	4.1	8.4	0	48	621
1	0.9	3.4	1.5	17.2	2.0	25.0	10	76	473
2	4.2	1.3	17.6	4.8	2.5	30.4	72	83	376
3	9.0	0.7	31.8	1.2	5.6	48.3	84	94	323
4	11.2	0.4	33.5	1.0	3.0	49.1	91	92	308

[a] Reaction conditions: birch sawdust (250 mg), catalyst (50 mg), H_2, 240 °C, 4 h, MeOH (10 mL).

[b] Based on the Klason lignin content of birch sawdust

[c] Based on the weight of dichloromethane extracted fraction (Klason lignin)

[d] 3MPa N_2

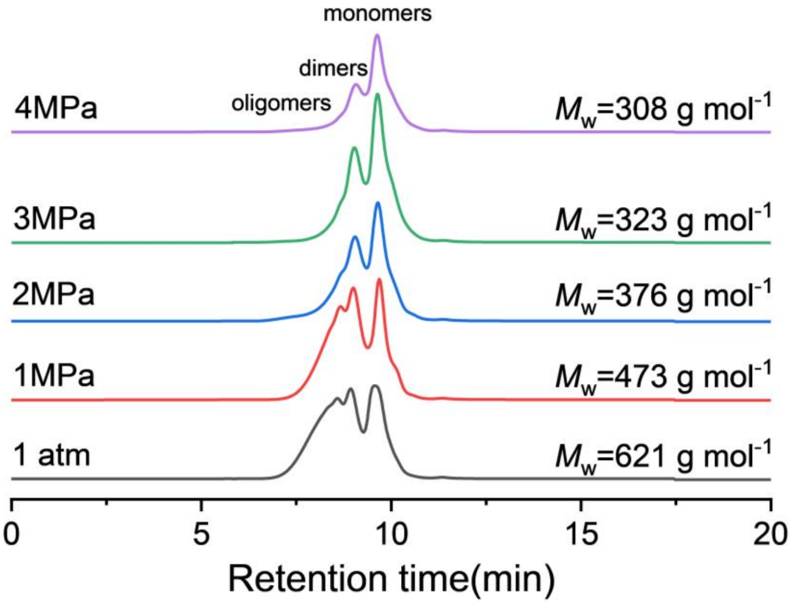

Figure S15. GPC spectra of lignin-derived products under different H_2 pressure. Reaction conditions: birch wood (250 mg), $Co_{0.15}$/N-C (50mg), MeOH (10 mL), H_2, 240 °C, and 4 h.

Table S9. Products distribution of $Co_{0.15}$/N-C-catalyzed hydrogenolysis of Birch with different catalyst dosage [a]

Catalyst	Monomers yield (wt%) [b]						Selectivity (mol%)	Delignification (wt%) [c]	M_w (g mol^{-1})
	**G1**	**G2**	**S1**	**S2**	Others	Total	**G1+S1**		
5	0.7	3.0	0.9	10.2	3.3	18.1	9	76	699
10	2.0	3.2	5.3	14.8	6.4	31.7	23	88	496
15	3.7	2.1	13.0	9.6	6.1	34.5	48	91	415
20	9.0	0.7	31.8	1.2	5.6	48.3	84	94	323
25	9.3	0.8	32.9	1.1	4.9	49.0	86	96	304

[a] Reaction conditions: birch sawdust (250 mg), $Co_{0.15}$/N-C, H_2 (3 MPa), 240 °C, 4 h, MeOH (10 mL).

[b] Based on the Klason lignin content of birch sawdust

[c] Based on the weight of dichloromethane extracted fraction (Klason lignin)

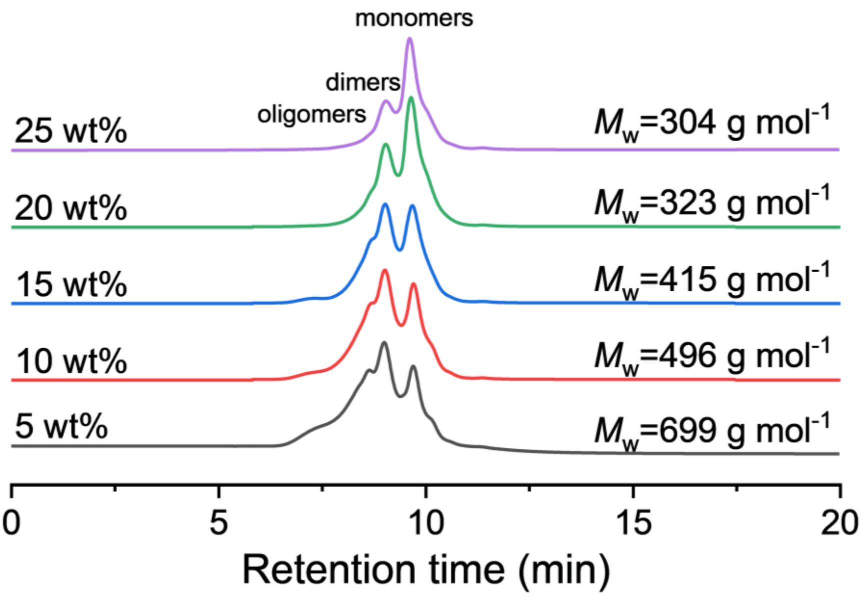

Figure S16. GPC spectra of lignin-derived products with different $Co_{0.15}$/N-C catalysts dosage. Reaction conditions: birch wood (250 mg), MeOH (10 mL), 240 °C, H_2 (3 MPa at 25 °C), and 4 h.

Table S10. Products distribution of $Co_{0.15}$/N-C catalyzed RCF reaction of various biomass. [a]

Biomass	Monomers yield (wt%) [b]						Selectivity (mol%)	Delignification (wt%) [c]	M_w (g mol^{-1})
	**G1**	**G2**	**S1**	**S2**	Others	Total	**G1+S1**		
Eucalyptus	7.3	2.0	24.1	3.3	4.9	41.6	75	90	370
Poplar	9.3	2.8	22.5	2.5	3.4	40.5	79	87	404
Spruce	11.8	1.5	ND [d]	ND	0.5	13.8	86	61	573
Pine	10.4	1.4	ND	ND	0.6	12.4	84	62	474
Miscanthus	6.1	0.6	5.7	0.4	13.3[e]	26.1	45	73	521

[a] Reaction conditions: substrate (250 mg), catalyst (50 mg), H_2 (3 MPa), 240 °C, 4 h, MeOH (10 mL).

[b] Based on the Klason lignin content of different sawdust.

[c] Based on the weight of dichloromethane extracted fraction (Klason lignin).

[d] N.D. refers to not detected.

[e] Containing two specific phenolic monomers (**H1**, 4.5 wt%; **G6**, 5.5 wt%) from the *p*-coumaric and ferulic acid units.

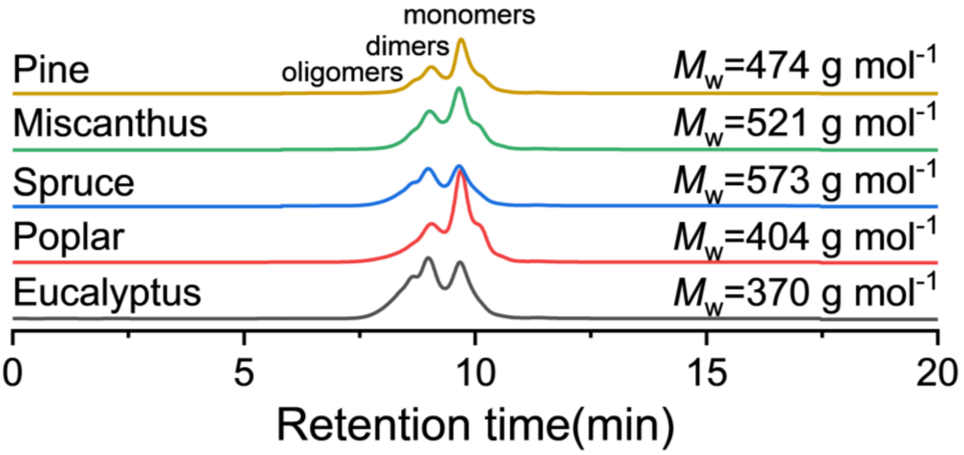

Figure S17. GPC spectra of lignin-derived products from various biomass over $Co_{0.15}$/N-C. Reaction conditions: substrate (250 mg), $Co_{0.15}$/N-C (50 mg), MeOH (10 mL), H_2 (3 MPa at 25 °C), and 4 h.

The reactivity of β-O-4 lignin mimics with $Co_{0.15}$/N-C

General experimental procedure: A 50 mL autoclave was contained with the lignin mimics (50 mg), $Co_{0.15}$/N-C (10 mg, 20 wt%), and MeOH (15 mL). The sealed autoclave was evacuated and charged with H_2 (3 MPa) at room temperature. After stirring at 240 °C for 4 h, the autoclave was allowed to cool to ambient temperature and then carefully depressurized. The oily product was obtained by filtering the reaction mixture and evaporating the filtrate. It was subjected to GC and GC-MS analysis after being dissolved in 5 mL of ethyl acetate solution containing a standard (tetradecane). The identification and quantification of products were assessed by comparison with authentic samples.

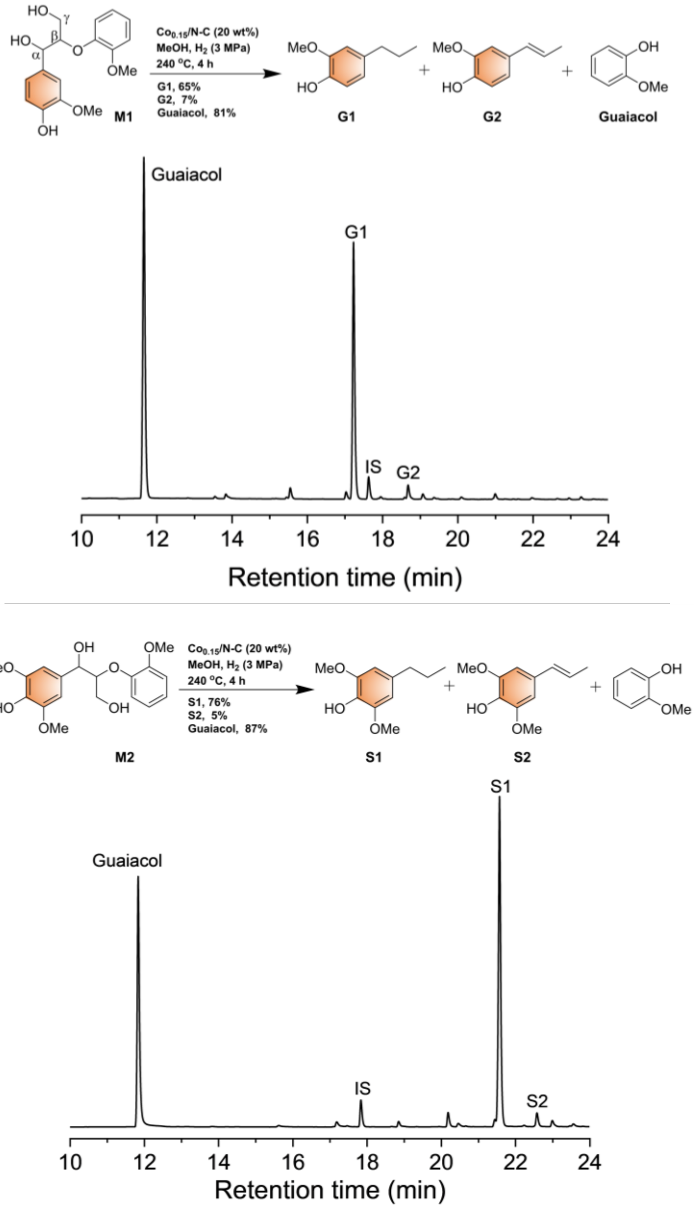

Figure S18. $Co_{0.15}$/N-C catalyzed hydrogenolysis of various lignin model compounds. M1 and M2 refer to phenolic β-O-4 dimers.

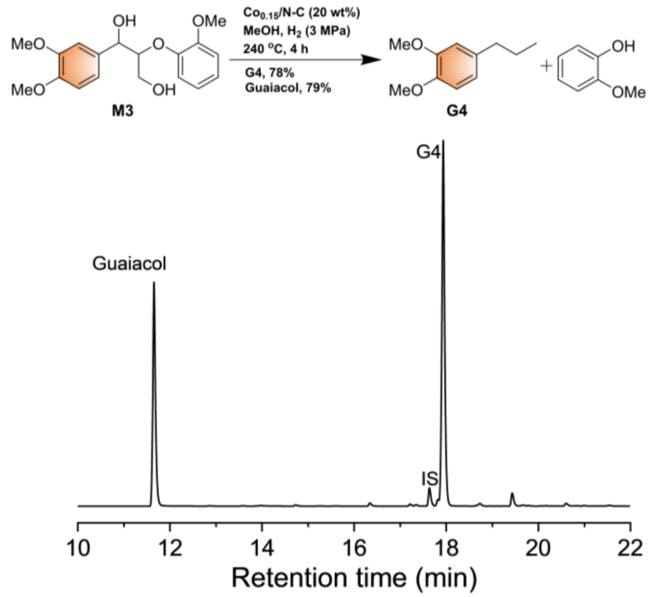

Figure S19. $Co_{0.15}$/N-C catalyzed hydrogenolysis of various lignin model compounds. M3 refers to nonphenolic β-O-4 dimers.

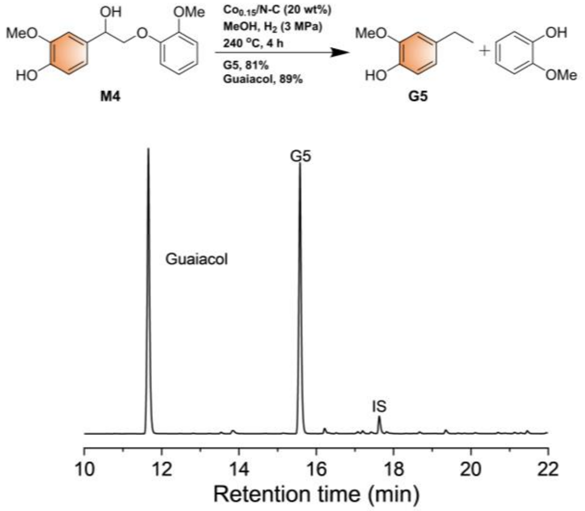

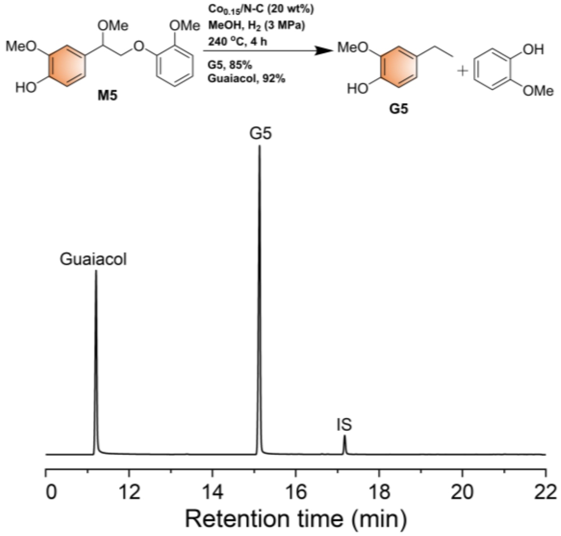

Figure S20. $Co_{0.15}$/N-C catalyzed hydrogenolysis of various lignin model compounds. M4 and M5 refer to β-O-4 dimers lacking of γ-CH_2OH unit.

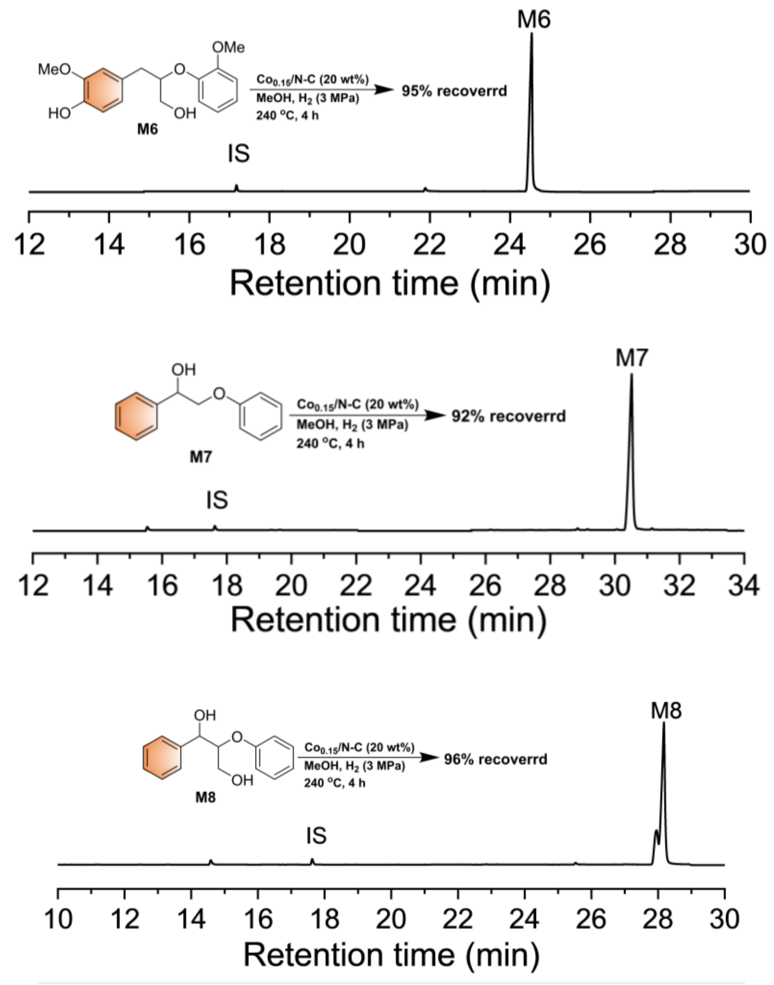

Figure S21. $Co_{0.15}$/N-C catalyzed hydrogenolysis of various lignin model compounds. M6, M7 and M8 refer to nonreactive β-O-4 dimer.

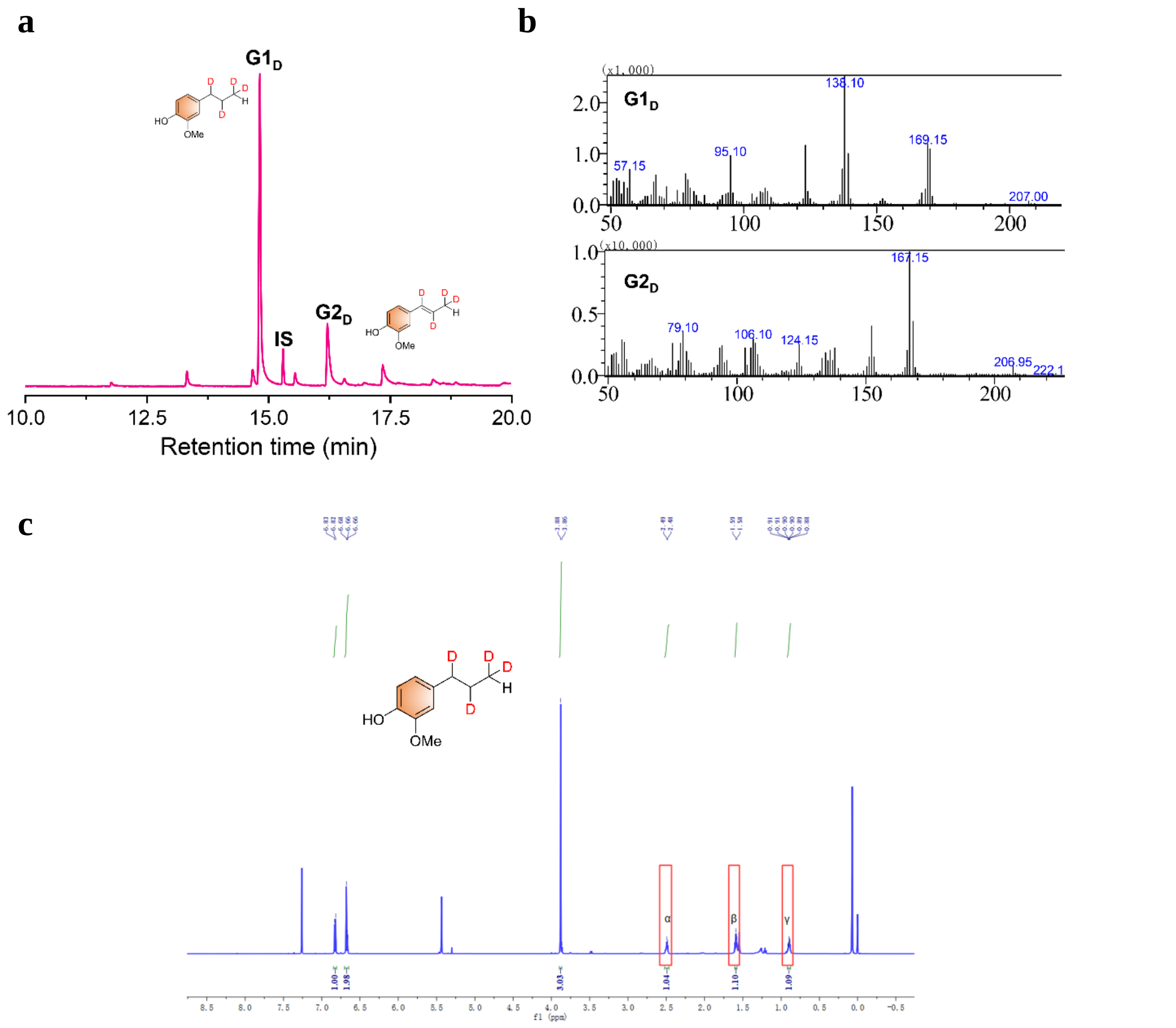

Figure S22. Hydrogenolysis of D-incorporated β-O-4 polymer LP-D with $Co_{0.15}$/N-C. (a) GC spectrum. (b) Mass spectrum derived from GC-MS. (c) ^1H NMR spectrum of isolated $G1_D$.

Table S11. Comparison of reductive catalytic fractionation results over different single-atom catalysts in the reported literature.

Catalyst	Monomer yields	Major products and selectivity		TON	Refs.
Pd/ZnO/C (Pd, 0.1 wt%)	40.7 wt%	4-propenyl phenols	62%	375	[3]
Ni@N-C SAC (Ni, 7.8 wt%)	31.2 wt%	4- propyl phenols	54%	6	[4]
Mo_1Al/MgO (Mo, 1.2 wt%)	46 mol%	methyl etherified coniferyl alcohol and sinapyl alcohol	92%	22	[5]
RuN/ZnO/C (Ru, 0.12 wt%)	46.4 wt%	4- propyl phenols	85%	431	[6]
Pt_1Ni/C (Pt, 0.3 wt%;)	43 wt%	4- propyl phenols	97%	166	[7]
$Pd_{0.25}/CN_x$ (Pd, 0.25 wt%)	52.7 C%	4- propyl phenols	71%	169	[8]
Co-phen/C (Co, 0.25 wt%)	34 wt%	4- propyl phenols	44%	100	[9]
$Co_{0.15}/N-C$ (Co, 0.15 wt%)	48.3 wt%	4- propyl phenols	84%	100	This work

Synthesis of epoxy monomers:

The monomer **G1** or **S1** (5.0 g, 30.1 mmol), ECH (6.97 g, 75.3 mmol), and TBAB (0.97 g, 3.01 mmol) were mixed in a round-bottomed flask and refluxed at 80 °C for one hour. Subsequently, a mixture of 20 wt% sodium hydroxide (0.48 g, 120.4 mmol) and TBAB (0.97 g, 3.01 mmol) was dropwise added to the flask under ice bath conditions, followed by a reaction at 30 °C for 0.5 h. The resulting **G1-PO or S1-PO** monomers were obtained through simple extraction, water removal, rotary evaporation and silica gel column chromatography.

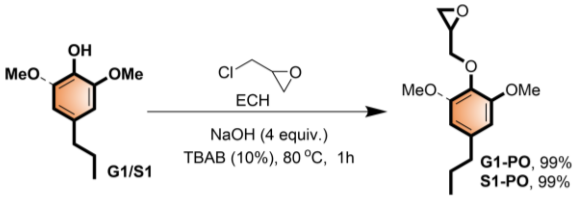

G1-PO, ^{1}H NMR (500 MHz, $CDCl_3$): δ 6.84 (d, J = 8.1 Hz, 1H), 6.76 - 6.65 (m, 2H), 4.19 (dd, J = 11.4, 3.5 Hz, 1H), 4.01 (dd, J = 11.4, 5.5 Hz, 1H), 3.85 (s, 3H), 3.37 (s, 1H), 2.87 (t, J = 4.5 Hz, 1H), 2.72 (dd, J = 4.7, 2.5 Hz, 1H), 2.52 (t, J = 7.6 Hz, 2H), 1.62 (dd, J = 15.0, 7.5 Hz, 2H), 0.93 (t, J = 7.3 Hz, 3H). ^{13}C NMR (126 MHz, $CDCl_3$) δ 149.40, 145.95, 136.6, 120.2, 114.36, 112.38, 70.47, 55.82, 50.23, 44.92, 37.63, 24.64, 13.77. MS (EI) found for $C_{13}H_{18}O_3$: 222.28.

S1-PO, ^{1}H NMR (500 MHz, $CDCl_3$): δ 6.38 (s, 2H), 4.08 (dd, J = 11.4, 4.2 Hz, 1H), 3.97 (dd, J = 11.4, 5.7 Hz, 1H), 3.82 (s, 6H), 3.42 - 3.28 (m, 1H), 2.79 (t, J = 4.5 Hz, 1H), 2.61 (dd, J = 4.9, 2.6 Hz, 1H), 2.51 (t, J = 7.7 Hz, 2H), 1.62 (dt, J = 14.8, 7.4 Hz, 2H), 0.94 (t, J = 7.3 Hz, 3H). ^{13}C NMR (126 MHz, $CDCl_3$) δ 153.02, 138.91, 105.40, 74.25, 56.11, 50.59, 44.88, 38.56, 24.66, 13.94. MS (EI) found for $C_{14}H_{20}O_4$: 252.31.

Synthesis of polyesters:

G1-PO or **S1-PO** (1.0 g, 4.5 mmol), PA (0.333 g, 2.25 mmol), PPNCl (2.58 mg, 0.0045 mmol), and DCU (2.0 mg, 0.009 mmol) were mixed in a 25 mL Schlenk flask. The reaction proceeded at 80°C under a nitrogen atmosphere for 12 hours. After cooling to room temperature, the mixture was dissolved in dichloromethane, precipitated into excess n-hexane, and subjected to multiple dissolution and precipitation cycles to remove unreacted monomers. The final polyester (poly(G1-PO-*alt*-PA) or poly(S1-PO-*alt*-PA)) was obtained through rotary evaporation and vacuum drying. The synthesis of copolyesters (poly(S1/G1-PO-*alt*-PA)) followed the same polymerization protocol, which involved using a specific molar ratio of **G1-PO** to **S1-PO** (**G1-PO**: **S1-PO** = 1: 4), with the same molar ratios of PA, PPNCl, and DCU as in the synthesis procedure described above.

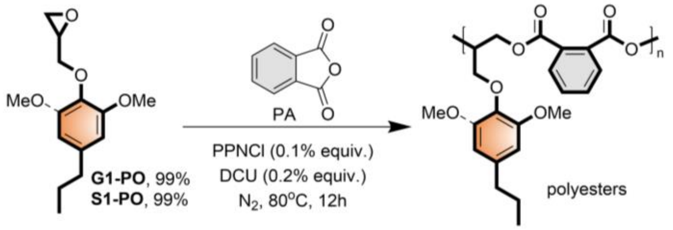

poly(G1-PO-*alt*-PA), ^{1}H NMR (500 MHz, DMSO-d_6) δ 7.71 - 7.51 (m, 4H), 6.82 (d, *J* = 7.4 Hz, 1H), 6.72 (d, *J* = 16.9 Hz, 1H), 6.56 (d, *J* = 6.4 Hz, 1H), 5.54 (s, 1H), 4.56 (s, 2H), 4.16 (d, *J* = 20.2 Hz, 2H), 3.64 (d, *J* = 15.2 Hz, 3H), 2.37 (s, 2H), 1.46 (d, *J* = 6.5 Hz, 2H), 0.90 - 0.70 (m, 3H). ^{13}C NMR (126 MHz, DMSO-d_6) δ 166.37, 166.18, 149.24, 145.54, 136.16, 131.70, 131.27, 131.21, 130.81, 128.86, 128.74, 120.09, 114.85, 112.75, 71.03, 67.42, 63.41, 55.45, 36.88, 24.15, 13.57.

poly(S1-PO-*alt*-PA), ^{1}H NMR (500 MHz, DMSO-d_6) δ 7.72 - 7.52 (m, 4H), 6.44 (s, 2H), 5.47 (s, 1H), 4.66 (s, 2H), 4.11 (s, 2H), 3.67 (d, *J* = 7.6 Hz, 6H), 2.45 (s, 2H), 1.55 (s, 2H), 0.87 (s, 3H).

poly(S1/G1-PO-*alt*-PA), ^{1}H NMR (500 MHz, DMSO-d_6) δ 7.63 (dd, *J* = 49.9, 28.4 Hz, 8H), 6.57 (ddd, *J* = 43.8, 35.4, 26.9 Hz, 5H), 5.49 (d, *J* = 53.1 Hz, 2H), 4.53 (dd, *J* = 67.5, 10.7 Hz, 4H), 4.21 (dd, *J* = 52.6, 23.3 Hz, 4H), 3.70 - 3.59 (m, 9H), 2.42 (s, 4H), 1.52 (s, 4H), 0.85 (s, 6H).

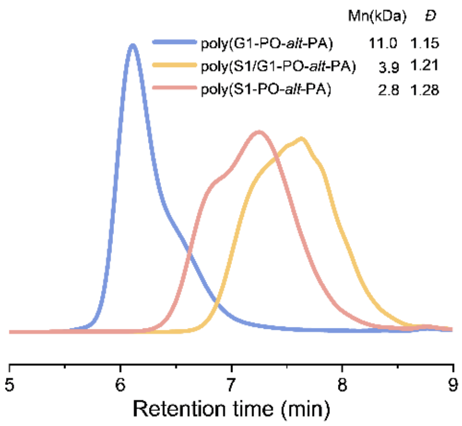

Figure S23. Molecular weight distributions of polyesters.

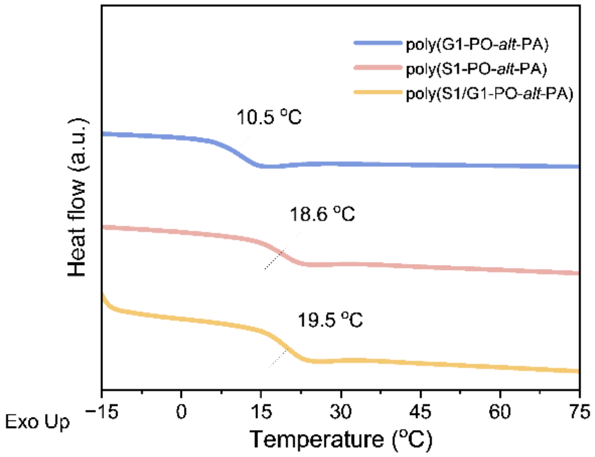

Figure S24. DSC curves of polyesters.

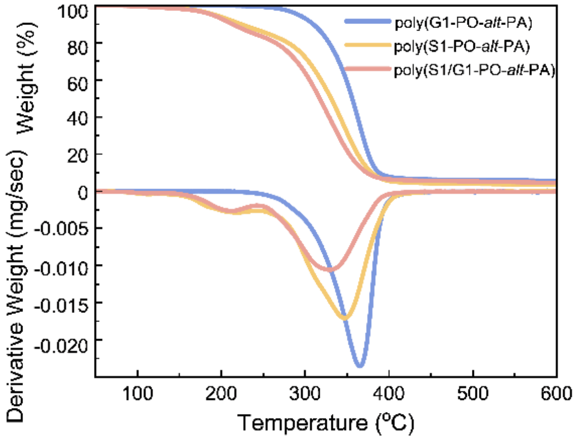

Figure S25. TGA and DTG curves.

Standard curves

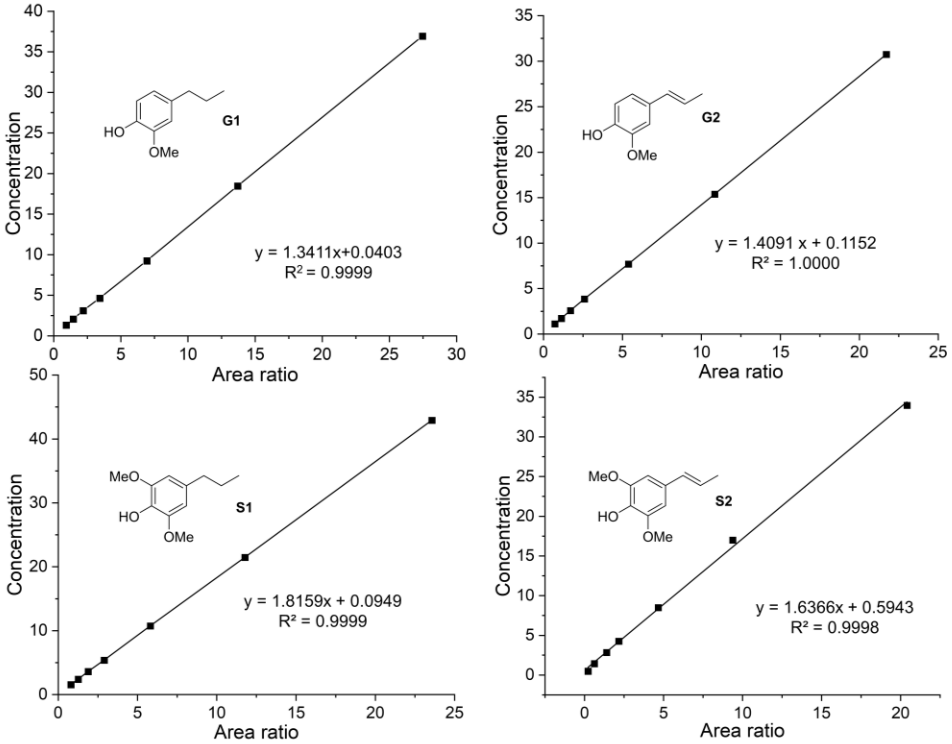

Figure S26. Standard curves of standard sample.

NMR spectra

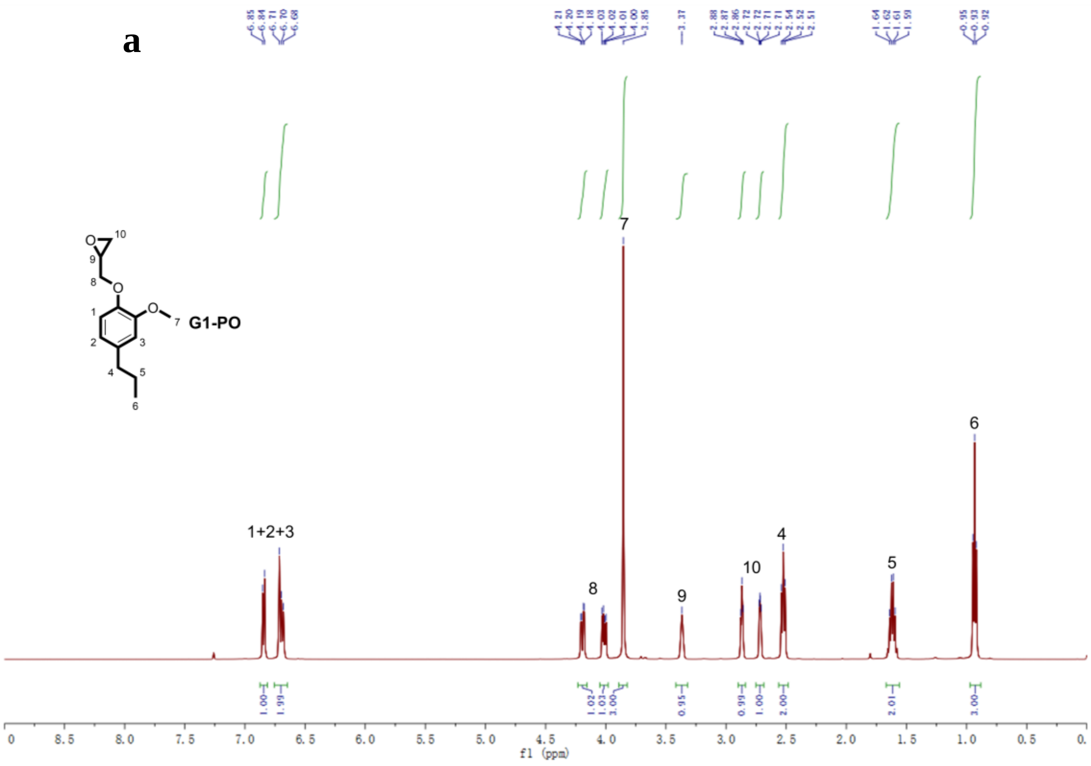

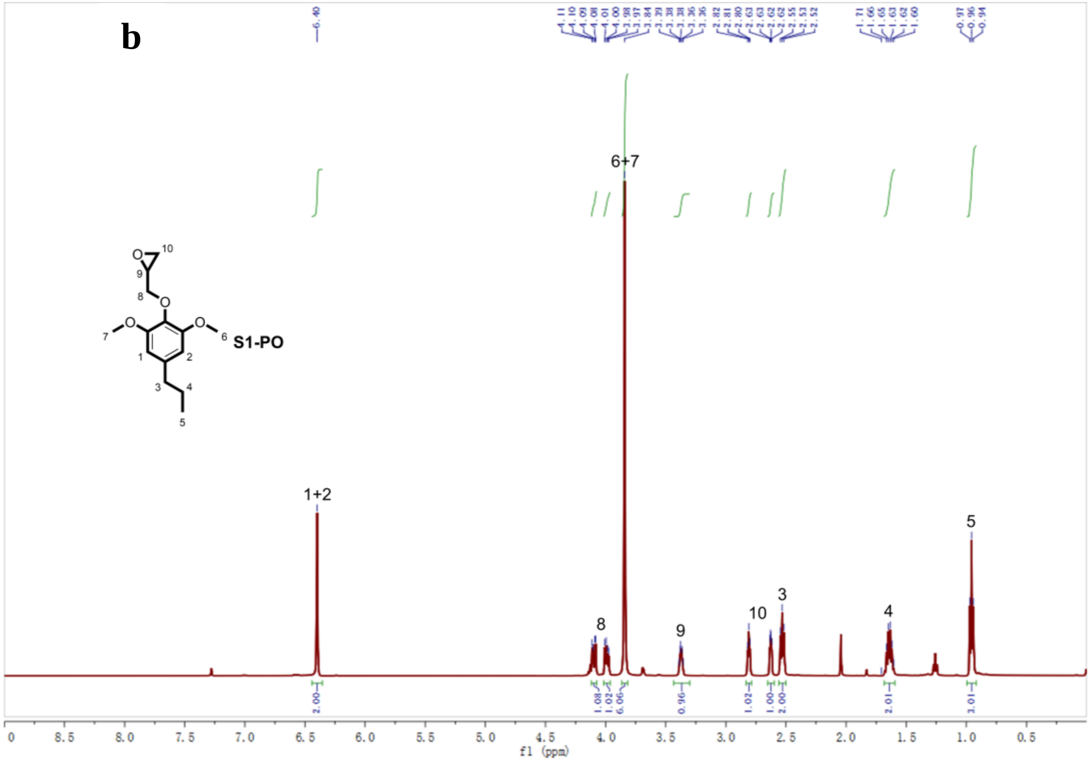

Figure S27. ^1H NMR spectra of G1-PO (a) and S1-PO (b).

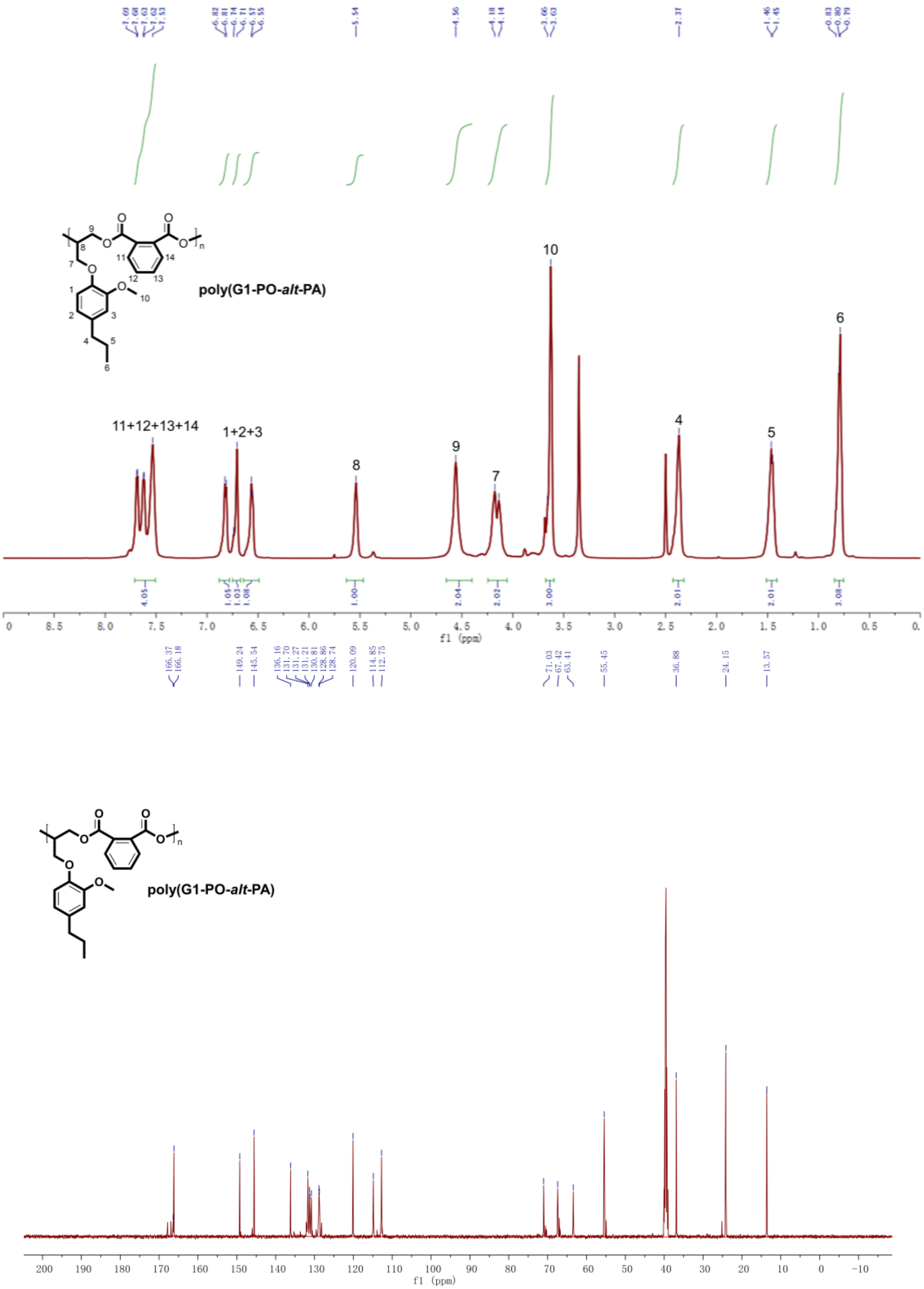

Figure S28. ^{1}H NMR, ^{13}C NMR spectra of poly(G1-PO-*alt*-PA).

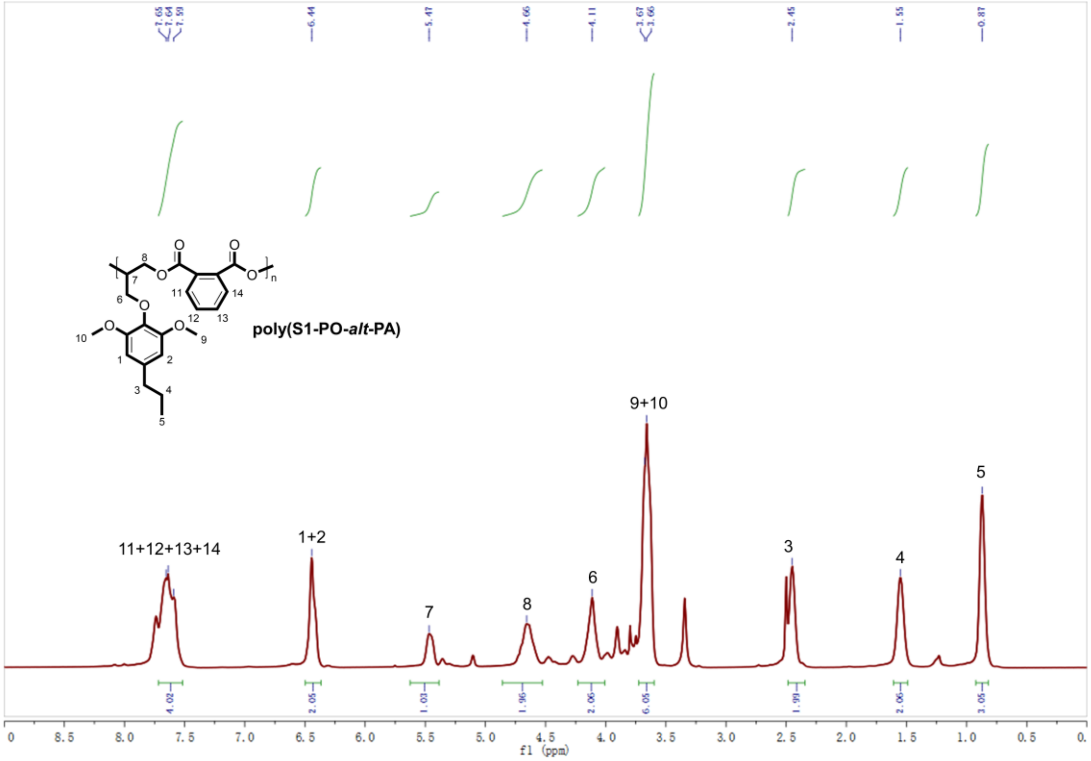

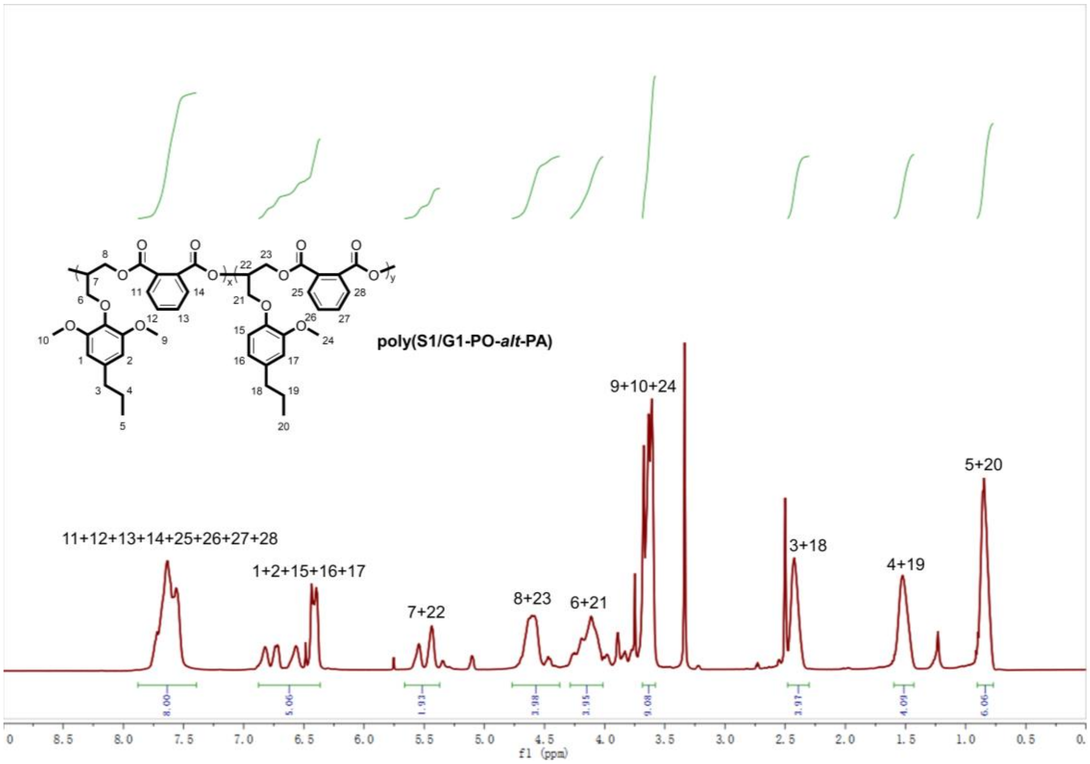

Figure S29. ^{1}H NMR spectra of poly(S1-PO-*alt*-PA), poly(S1/G1-PO-*alt*-PA).

References

[1] W. Chen, J. Pei, C. T. He, J. Wan, H. Ren, Y. Zhu, Y. Wang, J. Dong, S. Tian, W. C. Cheong, *Angewandte Chemie*. **2017**, *129*, 1630.
[2] B. Ravel, M. Newville, *Phys. Scr.* **2005**, 1007.
[3] S. Wang, X. Li, C. Fu, H. Li, G. Song, *ACS Catal.* **2024**, *14*, 3565.
[4] T. Li, B. Chen, M. Cao, X. Ouyang, X. Qiu, C. Li, *AIChE J.* **2023**, *69*, e17877.
[5] G. Meng, W. Lan, L. Zhang, S. Wang, T. Zhang, S. Zhang, M. Xu, Y. Wang, J. Zhang, F. Yue, *J. Am. Chem. Soc.* **2023**.
[6] Z. Liu, H. Li, X. Gao, X. Guo, S. Wang, Y. Fang, G. Song, *Nat. Commun.* **2022**, *13*, 4716.
[7] L. Chen, L. Pan, A. P. van Muyden, L. Bai, J. Li, Y. Tong, Z. Fei, A. Hagfeldt, G. Laurenczy, P. J. Dyson, *Cell Rep. Phys. Sci.* **2021**, *2*, 100567.
[8] J. Park, H. S. Cahyadi, U. Mushtaq, D. Verma, D. Han, K.-W. Nam, S. K. Kwak, J. Kim, *ACS Catal.* **2020**, *10*, 12487.
[9] S. Rautiainen, D. Di Francesco, S. N. Katea, G. Westin, D. N. Tungasmita, J. S. Samec, *ChemSusChem* **2019**, *12*, 404.
